# Genetic and Functional Analyses of Archaeal ATP-Dependent RNA Ligase in C/D Box sRNA Circularization and Ribosomal RNA Processing

**DOI:** 10.3389/fmolb.2022.811548

**Published:** 2022-03-25

**Authors:** Yancheng Liu, Yuko Takagi, Milyadi Sugijanto, Kieu Duong My Nguyen, Akira Hirata, Hiroyuki Hori, C. Kiong Ho

**Affiliations:** ^1^ Human Biology Program, University of Tsukuba, Tsukuba, Japan; ^2^ Biomedical Research Institute, National Institute of Advanced Industrial Science and Technology, Tsukuba, Japan; ^3^ Doctoral Program in Medical Sciences, Faculty of Medicine, University of Tsukuba, Tsukuba, Japan; ^4^ Department of Natural Science, Graduate School of Technology, Industrial and Social Science, Tokushima University, Tokushima, Japan; ^5^ Department of Materials Science and Biotechnology, Graduate School of Science and Engineering, Ehime University, Matsuyama, Japan

**Keywords:** circular RNA, RNA ligase, thermococcus kodakarensis KOD1, rRNA processing, C/D box sRNAs

## Abstract

RNA ligases play important roles in repairing and circularizing RNAs post-transcriptionally. In this study, we generated an allelic knockout of ATP-dependent RNA ligase (Rnl) in the hyperthermophilic archaeon *Thermococcus kodakarensis* to identify its biological targets. A comparative analysis of circular RNA reveals that the Rnl-knockout strain represses circularization of C/D box sRNAs without affecting the circularization of tRNA and rRNA processing intermediates. Recombinant archaeal Rnl could circularize C/D box sRNAs with a mutation in the conserved C/D box sequence element but not when the terminal stem structures were disrupted, suggesting that proximity of the two ends could be critical for intramolecular ligation. Furthermore, *T. kodakarensis* accumulates aberrant RNA fragments derived from ribosomal RNA in the absence of Rnl. These results suggest that Rnl is responsible for C/D box sRNA circularization and may also play a role in ribosomal RNA processing.

## Introduction

Numerous RNA molecules are involved in controlling gene expression to maintain cellular RNA metabolism. In addition to tRNA, mRNA and rRNA, cells also contain a striking diversity of additional RNA types, such as edited RNAs, circularized RNAs, trans-spliced RNAs, and other non-coding RNAs (Brennicke et al., 1999; Burroughs and Aravind, 2016; Kristensen et al., 2019). We hypothesize that RNA ligase, an enzyme that joins free RNA ends together, is a key player in producing a diverse set of RNAs by altering their structures. The recent finding that RNA ligase is responsible for generating a circular RNA molecule (circRNA) and could selectively modify the ends of the RNA raises the possibility that RNA ligase may also function to regulate cellular RNA metabolism.

ATP-dependent RNA ligase (Rnl) catalyzes the formation of phosphodiester bonds between the 5′-phosphate and 3′-hydroxyl termini of RNA (Uhlenbeck and Gumport, 1982). Rnl can join two single-stranded RNA molecules with or without a complementary bridging polynucleotide. It can also catalyze intramolecular ligation, leading to the formation of a covalently-closed circRNA. The biological functions of Rnl are firmly established in bacterial tRNA restriction/repair (Amitsur, 1987; Omari et al., 2006; Wang et al., 2007; Nandakumar et al., 2008), yeast and plant tRNA splicing (Sidrauski et al., 1996; Abelson et al., 1998; Englert and Beier, 2005), and kinetoplastid mitochondrial RNA editing pathways (McManus et al., 2001; Rusché et al., 2001; Schnaufer et al., 2001).

Many archaea species encode Rnl, and its structure is unique among polynucleotide ligases in that it forms a homodimeric quaternary structure. The crystal structure of *Pyrococccus abyssi* (PabRnl) and *Methanobacterium thermoautotrophicum* Rnl (MthRnl) have been solved and were shown to catalyze an intramolecular ligation of single-stranded RNA to form a covalently closed circRNA (Brooks et al., 2008; Gu et al., 2016). MthRnl can also transfer AMP to RNA containing 3′- phosphate termini to form 2′,3′-cyclic phosphate, and can selectively cleave adenosine from the 3′-hydroxyl end of the RNA, to form the 2′,3′-cyclic phosphate (Zhelkovsky and McReynolds, 2014; Yoshinari et al., 2017). Although the biological function of archaea Rnl is not known, RNA immunoprecipitation studies in *P. abyssi* suggest that it can interact with circular non-coding RNAs, including C/D box guide RNA (Becker et al., 2017).

Here we generated an allelic knockout of the Rnl gene in *Thermococcus kodakarensis* (TkoRnl; *TK1545*) and analyzed the change in RNA metabolism using high-throughput RNA-Seq technology. We showed that deletion of TkoRnl selectively dissipates circular C/D box sRNAs and other small RNA species. The conserved C/D box sequence elements were not strictly required for ligation activity of the archaeal Rnl. We also found that deletion of TkoRnl produces aberrant rRNA fragments, suggesting that TkoRnl may also participate in rRNA maturation process.

## Materials and Methods

### Strains, Media, and Culture Conditions

The *T. kodakarensis* KUW1 (Sato et al., 2005) and gene disruptant strain were cultivated under anaerobic conditions at 85°C (optimum growth temperature) in a nutrient-rich medium (ASW-YT) or a synthetic medium (ASW-AA). ASW-YT medium (1 L) contains 5 g yeast extract (Y) and 5 g tryptone (T) dissolved in artificial seawater (ASW) (Sato et al., 2003a). ASW-AA medium is a synthetic medium that contains a mixture of vitamins, modified Wolfe’s trace minerals, and the 20 canonical amino acids dissolved in 0.8 × ASW (Sato et al., 2003a; Atomi et al., 2004). Elemental sulfur (2 g) was added into 1 L ASW-YT and ASW-AA media before culturing. For all liquid media, resazurin (0.5 mg/L) was supplemented as an oxygen indicator, and 5.0% Na_2_S was added until the medium became colorless. For colony isolation, solid ASW-AA medium containing 1 g of Gelrite and 0.4 g of polysulfide per 0.1 L was used. The *E. coli* Mach1-T1 was used to construct the targeting plasmids and was grown at 37°C in LB medium containing ampicillin (100 mg/L). For RNA isolation and growth analysis, cells were cultured in MA-YT-P medium (0.8 × Marine Art SF1 reagent [Osaka Yakken Co. Ltd., Osaka, Japan], 5 g/L of yeast extract, 5 g/L of trypton, and 5 g/L of sodium pyruvate) which lacks elementary sulfur.

### Deletion of *TK1545* Gene

**FIGURE 1:**
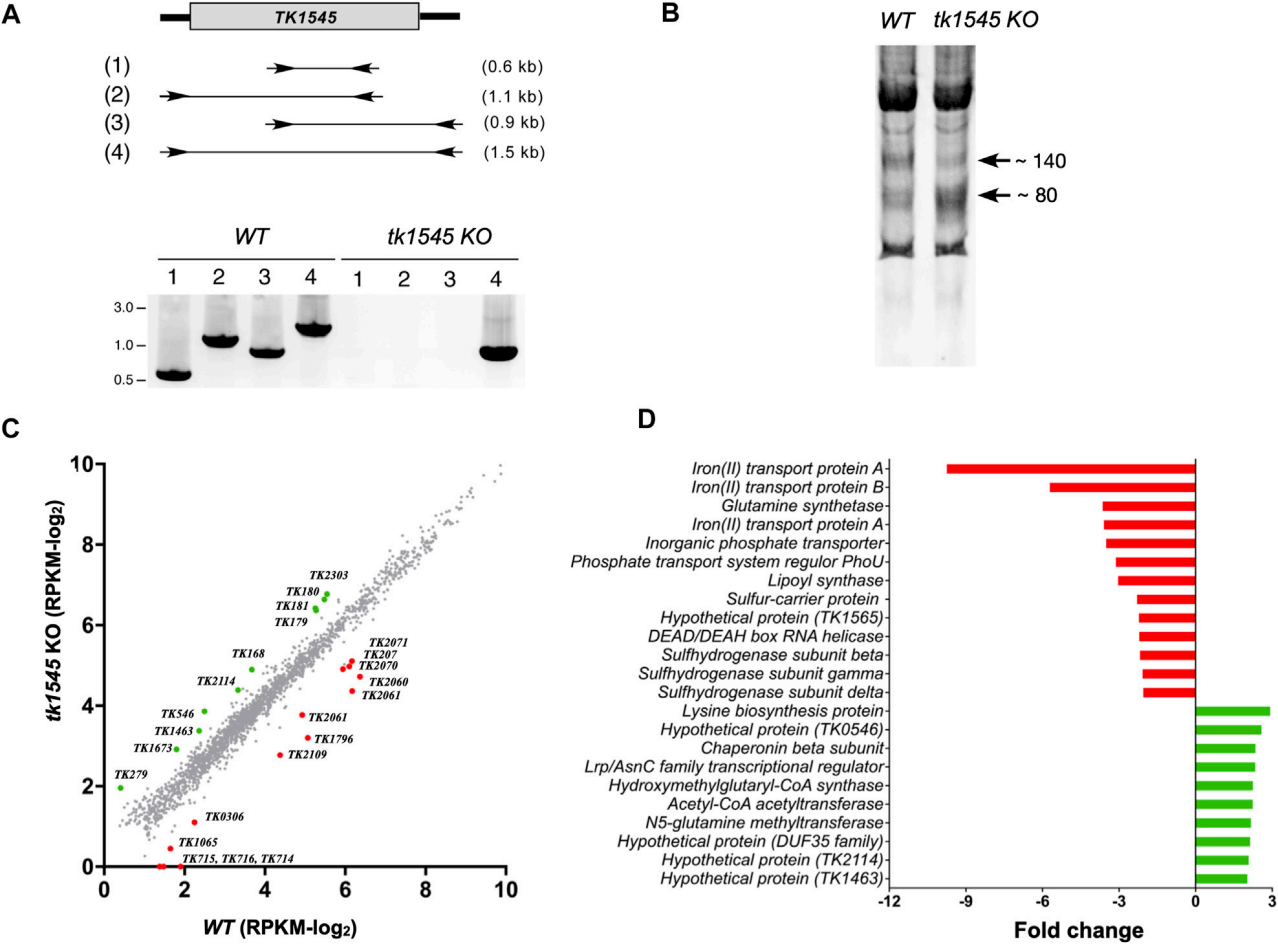
Differentially expressed genes by deletion of TkoRnl gene. **(A)** Generation of RNA ligase knockout in *T. kodakarensis*. The *TK1545* gene was replaced with the *pyrF* selectable marker (763 bp). The positions of four pairs of PCR primers and their expected product sizes are depicted in (1–4). The lower panel shows an agarose gel electrophoresis of PCR products derived from WT and *tk1545* KO genomic DNAs. **(B)** Total RNA extracted from WT and *tk1545* KO strains were separated using 10% denaturing polyacrylamide gel electrophoresis. Ethidium bromide staining of the gel is shown. **(C)** Transcriptosome analysis of WT and *tk1545* KO. Total RNA isolated from WT and *tk1545* KO cells were subjected to RNA-Seq analysis. Each dot represents an individual gene and are depicted according to read per million mapped reads (RPKM) values. The RPKM value with less than one RPKM were set to 0. Genes with RPKM of <2.0 in both WT and *tk1545* KO were omitted. Differentially expressed genes are depicted as colored dots. Green dots represent the 10 genes that are up-regulated, and red dots represent 13 genes that are down-regulated by more than 2-fold in *tk1545* KO (excluding the *tk1545* gene). The RPKM for *tk1545* from the WT and *tk1545* KO were 9.05 and 0.04, respectively. Genes that show greater than two-fold change are listed in **(D)** and in Supplementary Table S2.

A DNA fragment containing the *TK1545* gene along with its 5′- and 3′-flanking regions (∼1.0 kbp) was amplified with forward (5′ACT​CTC​TCC​TTT​TCT​CCA​ATT​TCG​G-3′) and reverse (5′-TCAGGATTTTGCAAA GTACTGACTGG-3′) primers using *T. kodakarensis* KUW1 genomic DNA as a template for PCR and was cloned into the Hinc II site of pUC118 to obtain pUC*-TK1545.* The *RNL* coding sequence in pUC*-TK1545* was replaced with a DNA fragment containing *pyrF* gene (*TK2276*: orotidine 5′-phosphate decarboxylase) and its promoter element as described (Sato et al., 2003b). This was accomplished by amplifying pUC*-TK1545* using two outward primers (5′-AGC​TGT​AAG​GGG​CCT​GTG​GAC​ATT​TC-3′ and 5′- GAT​ATC​ACC​GAG​AAG​AGT​GGG​AGC-3′) complementary to the upstream and downstream sequence of the *TkoRNL* coding sequence. The amplified plasmid fragment was ligated with a PvuII-PvuII restriction fragment (763 bp) containing the *pyrF* marker gene derived from pUD2 plasmid (Sato et al., 2003b). All the sequences of the inserted region were verified with DNA sequencing. The resulting targeting plasmid, with *pyrF* marker gene inserted between the 5′- and 3′-flanking regions of *RNL* gene, was used to transform *T. kodakarensis* KUW1 strain. Cells grown in ASW-YT-S0 medium at 85°C for 10 h were harvested and suspended in 200 μL of 0.8 × ASW and kept on ice for 30 min. Then, 3 μg of the plasmid was gently added into the suspended cells and kept on ice for 1 h. Transformants were cultivated in an uracil-free ASW-AA-S0 at 85°C for 40 h. Next, 200 μL of the culture was transferred to a fresh medium and cultivated under the same conditions to enrich transformants displaying uracil prototrophy. The cultures (100 μL) were spread onto ASW-AA-S0 solid medium and incubated at 85°C for 3 days. Only cells that obtained a phenotype exhibiting uracil prototrophy by homologous recombination can grow in the absence of uracil. Single colonies were selected and then cultured in ASW-YT-S0 medium at 85°C for 10 h. Throughout this article, the term wild-type (WT) refers to KUW1 and *tk1545* KO refers to TkoRnl deletion in the KUW1 strain. The cells of the *tk1545* KO strain were harvested and suspended in distilled water. Genomic DNA was extracted from the cells using phenol-chloroform treatment. The replacement of *TK1545* gene with *pyrF* gene was verified with PCR (Figure 1A).

### RNA Isolation and RNA-Seq Analysis

*T. kodakarensis* WT and *tk1545* KO (1.6 L) were cultured in MA-YT-P medium at 85°C and harvested when the absorbance at 660 nm reached ∼0.7 (late-log phase). Total RNA was isolated using TRIzol reagent (Invitrogen) following the manufacture’s instruction. The total RNA and small RNA libraries were prepared using standard Illumina protocol by UB Genomics and Bioinformatics Core at The State University of New York (SUNY) at Buffalo, United States. The high-throughput RNA-sequencing was performed using Illumina HighSeq2500 technology (51-base; pair-ended read). Raw reads were trimmed, and FastQC was used to determine the clean reads. For both WT and *tk1545* KO datasets, 80 percent of the reads had Phred quality scores >35. The reads were mapped to *T. kodakarensis* reference genome (NC_006624.1) using bowtie2-2.2.9 with the option preset set to sensitive and alignment type set to local mode. The reads per kilo-base per million mapped reads (RPKM) were calculated using featureCount (Liao et al., 2014). From the WT dataset, 101 million reads were obtained, of which 94.86% mapped to the reference genome, with a median RPKM of 9.8. From the *tk1545* KO dataset, 58 million pairs of reads were obtained, of which 94.54% were mapped to the reference genome with a median RPKM of 10.9 (Supplementary Data S1).

For the small RNA-Seq, the 5′-adaptor sequence (5′-GTT​CAG​AGT​TCT​ACA​GTC​CGA​CGA​TC) and 3′-adaptor sequence (5′-TGG​AAT​TCT​CGG​GTG​CCA​AGG) were trimmed using Cutadapt, with 80 percent of the reads having Phred quality scores of >35. The reads aligned to reference genome as described above. We obtained 22 million pairs of reads from the WT sample, of which 79.94% mapped to the reference genome. From the *tk1545* KO sample, 16 million reads were obtained, and 81.43% mapped to the genome (Supplementary Data S1).

### Analysis of circRNA from RNA-Seq Data

A custom Perl script was used to identify RNA-Seq reads containing circular junction sequences. The screening was done to identify RNA-Seq reads containing two segments of at least 20-nts matching the reference genome; the two matched segments should be encoded in the same direction but inverse order in the reference genome. From total RNA Seq data, 410,073 and 281,828 reads containing circular junctions from the WT and *tk1545* KO were identified, respectively (Supplementary Data S2). To reduce redundancy, reads containing similar junction sequences within five nucleotide variations were classified into the same group, which decreased the number of candidate circRNA reads to 12,632 for WT and 9,744 for *tk1545* KO. Subsequently, circRNA junction reads with less than 100 independent read counts were eliminated. This criterion identified 113 and 63 reads containing circRNA junction sequences from WT and *tk1545* KO, respectively. A similar strategy was used to identify circRNAs from the small RNA-Seq datasets, except that circRNA reads predicted to be longer than 10 kb and read counts of less than 20 were eliminated (Supplementary Data S3).

### Detection of circRNA Using RT-PCR

We performed RT-PCR to detect predicted circular RNAs. Primers containing gene-specific sequences were used for reverse transcription reaction. The reaction mixture (40 μL) containing total RNA (200 ng) from either WT or *tk1545* KO was incubated with 25 μM gene-specific primer, 0.5 mM dNTP, and 50 U ReverTra Ace-α (Toyobo, Japan) in a supplied reaction buffer at 55°C for 10 min (for primer sequences, see Supplementary Table S1). The reaction was terminated at 95°C for 10 min, and an aliquot (1 μL) was used as a template for PCR. PCR (50 μL) contained 0.2 μM circular junction primer and gene-specific primer, 2.5 U Paq5000 DNA polymerase (Toyobo, Japan) programmed for 25 cycles (95°C for 30 s; 60°C for 30 s; 72°C for 10 s). PCR products were separated on 3% low-range ultra-agarose gel, stained with ethidium bromide, and visualized using UV.

### RNA Ligase and RNA Substrates

His-tagged MthRnl and His-tagged T4 RNA Ligase 2 (T4 Rnl2) were produced in *E. coli* and purified from soluble bacterial extracts using Ni-agarose chromatography as described previously (Ho and Shuman, 2002; Torchia et al., 2008). *In vitro* transcription was used to synthesize RNAs from PCR amplified linear DNA templates containing a T7 RNA polymerase promoter. RNAs containing 5′ triphosphate were purified by electrophoresis through a non-denaturing 8% polyacrylamide gel. The RNAs were then treated with calf intestinal alkaline phosphatase, extracted by phenol-chloroform, and ethanol precipitated. The *in vitro* transcribed RNAs and the chemically synthesized 24-mer RNA were labeled at the 5′-end with [γ-^32^P] ATP using T4 polynucleotide kinase and purified on the non-denaturing polyacrylamide gel.

### Cloning of Small RNA Fragments

Total RNA (15 µg) from WT and *tk1545* KO were separated using 10% denaturing polyacrylamide gel, and RNA ranging from 70 to 120 nt was isolated by elution from the gel with Tris-EDTA (TE) buffer. RNA was ethanol-precipitated and resuspended to 50 µL with TE buffer. The isolated small RNA was used for the adapter-mediated RNA cloning with SOLiD Small RNA Expression Kit (Ambion). Ligation and reverse-transcription were performed according to the manufacturers’ instructions. cDNA was amplified using Taq DNA polymerase, inserted into TOPO cloning vector (Invitrogen), and transformed into *E. coli* DH5α. The region of cDNA insertion within the TOPO vector was PCR amplified directly from the bacterial colonies, and product size was analyzed on 1.8% agarose gel. The majority (90%) of the plasmid recovered contained primer-dimer insert. Therefore, PCR product corresponding to >40 bp cDNA insert was regarded as a “positive” clone and was sequenced to reveal the identity of cloned RNA species. PCR product length corresponding to <40 bp insert was omitted, as it likely represented a fragment derived from a primer dimer.

## Results

### Deletion of *T. kodakarensis* Rnl Gene

*TK1545* encoding for *T. kodakarensis* ATP-dependent RNA ligase (TkoRnl) was removed via homologous recombination using a non-replicating targeting vector carrying orotidine-5′-monophosphate decarboxylase gene (*pyrF*) flanked by ∼700 bp of upstream and downstream *TK1545* DNA sequence. The plasmid was transformed into *T. kodakarensis* strain KUW1 (∆*pryF*) and the *Ura* ^*+*^ transformants were recovered. Deletion of *TK1545* locus was confirmed using PCR analysis (Figure 1A). Expression of *TK1545* was verified using RNA-Seq analysis, as described below. No significant difference in growth phenotype was observed between the WT and *tk1545* KO strains in nutrient-rich medium, at optimal (85°C) and elevated temperatures (93°C) (Supplementary Figure S1), which implies that TkoRnl is not essential for viability. Polyacrylamide gel electrophoresis analysis of the small RNA population suggested that the relative distribution of short RNA species, ranging from 80 to 140 nt, differed significantly between the two strains (Figure 1B). Compared to the parental strain, the level of ∼140-nt species was reduced while that of the ∼80-nt species was increased in the *tk1545* KO strains. Therefore, both total and small RNA-Seq analyses were performed to evaluate the physiological consequences of TkoRnl deletion*.*

### Transcriptome Analysis

Total and small RNAs isolated from the wild-type and *tk1545* KO strains were sequenced on an Illumina HighSeq platform sized at 51-bp and mapped uniquely to the annotated genome. The gene expression abundance was normalized using RPKM (Supplementary Data S2), and scatterplots were used to assess the expression variation of the genes between the WT and *tk1545* KO from the total RNA-Seq dataset (Figure 1C). Finally, 23 genes were identified that showed altered expression changes of >2-fold, of which 13 were up-regulated, and 10 were down-regulated in *tk1545* KO compared to the WT (Figure 1D; Supplementary Table S2). Many of these genes that exhibit differential gene expression are encoded on the same polycistronic transcription unit; iron transport proteins (*TK0714*, *TK0715*, and *TK0716*)*,* phosphate transporter proteins (*TK2060* and *TK2061*), sulfur reductase subunits (*TK2071* and *TK2072*), and Acetyl-CoA acetyltransferase pathway (*TK0179*, *TK0180*, and *TK0181*). The genes encoded within each operon were either enhanced or reduced to a similar extent in *tk1545* KO. Transcriptome analysis from the small RNA-Seq data is shown in Supplementary Figure S2.

### Computational Predictions of Circular RNAs

It has been widely reported that non-coding RNAs, including tRNAs, C/D box sRNA, and rRNA processing intermediates are circularized in archaea (Tang et al., 2002; Starostina et al., 2004; Danan et al., 2012; Randau, 2012; Su et al., 2013; Becker et al., 2017). We hypothesize that if Rnl is responsible for generating circRNAs, we would identify its RNA target by comparing the circRNA reads obtained from WT and *tk1545* KO. Our criteria for detecting circular reads from the RNA-Seq data were as follows: 1) the 51-nts RNA-Seq reads containing two segments, and each segment has a minimum 20-nts match to the reference genome sequence; 2) the two matched segments within the read are encoded in the same transcriptional direction, but are positioned in inverse order in the reference genome (Danan et al., 2012); and 3) the two matched segments are fused to form a unique circular junction sequence. Variation in the circular junction within five nucleotides in the locus was classified into the same group to reduce redundancy (Supplementary Data S2). The predicted length of individual circRNA was deduced from the distance between the two homologous segments in the genome reference. For the total RNA-Seq data, we selected reads that support more than 100 counts (Supplementary Data S2). For the small RNA-Seq data, we selected read supported more than 20 counts and did not include reads that were predicted to be longer than 10 kb (Supplementary Data S3).

In the WT *T. kodakarensis,* 31 circRNA reads were detected from the small RNA Seq data set, many of which were derived from C/D box sRNAs (Supplementary Table S2). The C/D box sRNA molecule has four sequence elements: the C box and C’ box motifs with the consensus sequence RUGAUGA, and the D box and D’ box motifs with the consensus sequence CUGA. Of the 61 putative *T. kodakarensis* C/D box sRNAs we identified, 26 C/D Box sRNAs had circRNA reads (Supplementary Table S3). Analysis of *P. abyssi* RNA-Seq dataset detected 24 circRNA reads (Toffano-Nioche et al., 2013), many of which were shown to be circularized (Becker et al., 2017)*.* Other circRNA reads detected from *T. kodakarensis* include protein coding genes (*TK0058* [HAD superfamily hydrolase], *TK2034* [Universal stress protein], *TK2109* [lipoyl synthase], *TK0894* [hypothetical protein], *TK1980* [ferredoxin oxidoreductase, alpha subunit], *TK0135* [ferredoxin oxidoreductase, beta subunit]) and non-coding RNAs designated here as ncRNA01, ncRNA02, and ncRNA03 (Table 1; Supplementary Data S2). Some of these RNAs were previously reported as C/D box sRNA (Jäger et al., 2014). CircRNAs were also detected in abundance from rRNA operon and tRNA^Trp^ as previously reported (Danan et al., 2012).

**TABLE 1:** List of circRNAs in *T. kodakarensis*. The top part of the table shows list of circRNAs that were detected in WT but were either absent or significantly reduced in *tk1545* KO small RNA-Seq dataset. The bottom part of the table shows a list of circRNAs that were detected in both WT and *tk1545* KO. The value in parenthesis shows the number of reads detected in total RNA-Seq dataset.

Locus/CircRNA name	Circular junction (+/− 5 nucleotides)	Predicted length^a^	*T. kodakarensis* (WT)		*T. kodakarensis* (*tk1545* KO)		Alias and predicted transcription start site^b^	
			Number of aligned read	Number of circular reads	Number of aligned read	Number of circular reads		
sR01	47786 to 47847	62	2,167	395	1,776	7	TKOc and Sno. 19	47910
			(368)	(18)	(193)	(0)		
sR05	116401 to 116466	66	445,142	354	333,035	0	Tko-sR07	116468
			(531)	(54)	(286)	(0)		
sR13	279797 to 279863	67	59,144	47	58,048	0	Tko-sR14	279865
			(142)	(2)	(62)	(0)		
sR15	316130 to 316191	62	1,629,229	698	1,523,609	8	Tko-sR16	316202
			(611)	(113)	(352)	(0)		
sR20	558818 to 558879	62	1,622	21	1,400	0	Tko-sR20	558914
			(84)	(46)	(37)	(0)		
sR28	832364 to 832425	62	65,322	72	54,801	1	Tko-sR26	832424
			(340)	(123)	(156)	(0)		
sR29	940146 to 940209	64	475,600	36	283,215	0	Tko-sR29	940134
			(551)	(11)	(335)	(0)		
sR31	963853 to 963919	67	562,648	4,655	721,884	34	Tko-sR31	963842
			(252)	(343)	(11)	(1)		
sR34	1103565 to 1103626	62	20,161	119	19,347	0	Tko-sR35	1103625
			(797)	(14)	(478)	(0)		
sR37	1159583 to 1159644	62	356,961	44	195,173	1	Tko-sR37	1159732
			(139)	(21)	(94)	(0)		
sR38	1167276 to 1167338	63	11,287	106	4,940	0		
			(111)	(3)	(94)	(0)		
sR41	1226838 to 1226899	62	1,967	31	610	0	Tko-sR41	1226903
			(359)	(202)	(19)	(0)		
sR42	1226948 to 1227017	70	12,085	7,194	17,658	10	Tko-sR42	1226948
			(226)	(145)	(83)	(0)		
sR46	1371729 to 1371790	62	176,232	188	79,569	3	Tko-sR50	1371720
			(540)	(258)	(118)	(3)		
sR49	1446209 to 1446268	60	62,328	358	36,039	1	Tko-sR52	1446266
			(224)	(376)	(104)	(0)		
sR52	1476851 to 1476917	67	28,701	467	42,613	6	Tko-sR54	1476850
			(349)	(4)	(208)	(0)		
sR58	1947796 to 1947856	61	926	95	845	0		
			(66)	(5)	(52)	(0)		
sR61	2070055 to 2070116	62	100,212	92	31,284	0	Tko-sR67	2070114
			(235)	(22)	(188)	(0)		
ncRNA01	1053772 to 1053834	63	1,267,983	68	587,087	3	Tko-sR33	1053770
			(198)	(48)	(113)	(0)		
ncRNA02	1257020 to 1257081	62	8,320	139	6,342	0	TKOcandSno66	1257078
			(42)	(135)	(3)	(0)		
ncRNA03	1593064 to 1593134	71	7,565	403	9,633	1		
			(149)	(4)	(83)	(0)		
*TK0058*	51411 to 51477	67	1,816	589	780	1	Tko-sR01	51477
			(535)	(493)	(194)	(1)		
*TK2034*	1826865 to 1826930	66	771	66	477	2	Tko-sR22^*c*^	1826865
			(159)	(22)	(84)	(0)		
*TK2109*	1894519 to 1894579	61	14,240	28	11,134	0		
			(1,092)	(857)	(164)	(4)		
tRNA-Trp	1945728 to 1945789	62	28,907	2,023	23,156	1,292		
			(3,195)	(1,415)	(1,680)	(987)		
*TK0135*	108461 to 116468	8,008	457,060	90	576,950	110		
			(1,94,213)	(0)	(114,857)	(0)		
*TK0894*	779653 to 779796	144	317	28	215	28		
			(1,487)	(0)	(1,207)	(0)		
*TK1980*	1784662 to 1785579	918	1,322	42	739	53		
			(104,981)	(0)	(72,305)	(0)		
16S RNA-c1	2022801 to 2024382	1,582	404,505	96,319	268,720	53,186		
			(55,116,954)	(6,836)	(30,650,056)	(4,733)		
23S RNA-c1	2024584 to 2027631	3,048	867,077	27,733	610,944	11,628		
			(109,488,453)	(282,955)	(62,941,809)	(205,719)		
23S RNA-c2	2024595 to 2027605	3,011	867,051	83	610,939	35		
			(109,486,015)	(42,016)	(62,940,686)	(23,246)		

a Position of circular junction and predicted length of each circRNAs, were determined by mapping to the T. kodakarensis reference genome (See Supplementary Data S3).

b The alias and predicted start site of transcription are from (Jäger et al., 2014).

c Annotated as sR22 in (Jäger et al., 2014).

### *TK1545* is Required for C/D Box sRNA Circularization

**FIGURE 2:**
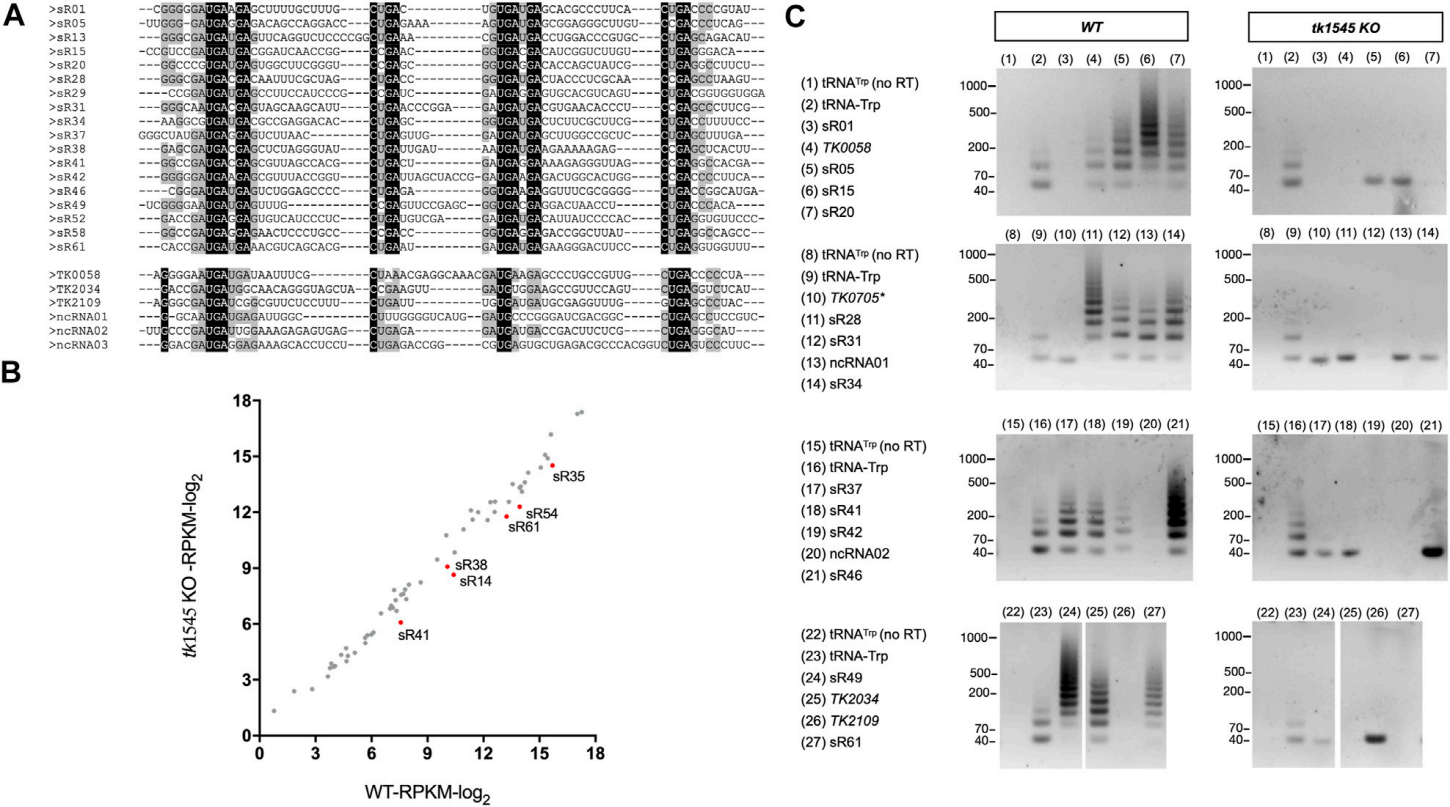
Effect on RNA circularization by *TK1545* knockout. **(A)** Sequence alignment of circRNAs which were reduced or absent in *tk1545* KO (see Table 1). Putative circular C/D box RNAs are listed on top and other circRNAs are listed on the bottom. The positions of conserved motifs (C box, C′ box, D box, and D′ box) are indicated. Identical nucleotides are highlighted in black with white text and conserved nucleotides are highlighted in gray. **(B)** Expression of C/D box RNA. Expression analysis of sixty-one *T*. *kodakarensis* C/D box RNA listed on Supplementary Table S3. Red dots represent the six C/D box RNAs reduced by more than 2-fold in *tk1545* KO. **(C)** RT-PCR analysis was used to detect circRNAs from total RNA isolated from WT and *tk1545* KO. The primer set used to detect circular RNA species is indicated on the right of the gel. A circular tRNA^Trp^ intron primer was used as a positive control (tRNA-Trp). As a negative control, assay was performed without reverse transcriptase treatment using tRNA^Trp^ intron primers [tRNA^Trp^ (no RT)], or with the *TK0705* primers (Gene ID 3234407: NADP-dependent glyceraldehyde-3-phosphate dehydrogenase, which is not circularized in both WT and *tk1545* KO). The size of the molecular weight markers is indicated on the left. PCR product that migrates ∼40 nts most likely represented a product derived from a primer dimer.

Analysis of *tk1545* KO RNA-Seq data revealed that 24 out of 31 circRNAs that were detected in WT were either absent or significantly reduced in *tk1545* KO (Table 1). These included 18 C/D box sRNAs, three non-coding RNAs, and three protein-coding genes (*TK0058*, *TK2109*, and *TK 2034*)*.* Similar results were obtained when RNA-Seq analysis of WT and *tk1545* KO were analyzed on a SOLiD sequencing platform (Supplementary Table S4) (Liu, 2022). Figure 2A shows a sequence alignment of circRNAs that were affected in *tk1545* KO. All these circRNAs have a similar length (61–71 nts) and homology to the C/D box sRNA. The terminal ends are generally GC-rich, and sequences at the termini can hybridize to form a stem, a structural characteristic found in C/D box sRNA. Secondary structure analysis suggests that hybridization between the two terminal ends could be critical for RNA circularization (Supplementary Figure S3). The majority of the RNAs that were circularized (21 out of 24 shown in Figure 2A) could potentially form three or more base pairings to form a terminal stem (Supplementary Figure S3A). In contrast, 16 out of 17 non-circular C/D box sRNAs are less likely to form a terminal stem with two or less base pairings (Supplementary Figure S3B). We note that relative abundance of six C/D box sRNAs (sR14, sR35, sR38, sR41, sR54, and sR61) out of sixty-one C/D box sRNAs that we identified, were reduced by 2-fold or more in *tk1545* KO compared to the WT (Figure 2B).

There were no significant changes in the level of circRNA reads derived from tRNA^Trp^ intron and 16S and 23S rRNAs. We note that circularization of *TK0135*, *TK0894* and *TK1980*, all of which have predicted circRNA size of >100 nts, were not significantly affected by TkoRnl deletion. Circular *TK0894, TK1980* and *TK0135* were not detected in the whole RNA-seq data (Table 1), suggesting that these RNAs may likely circularize after the degradation or processing of the transcript into small RNA.

RT-PCR analysis was performed to verify whether the circular RNA species are present in *T. kodakarensis* (Figure 2C). In this procedure, reverse transcription primer was designed to hybridize the gene specific sequence. If this primer hybridizes to circular RNA, reverse transcription will generate a long “rolling-circle” single-stranded cDNA. Subsequent PCR with circular junction and gene specific primers generate a ladder of DNA fragments, which can be visualized on the gel electrophoresis (Starostina et al., 2004). A tRNA^Trp^ primer was used as a positive control because tRNA^Trp^ introns accumulate circRNA reads in both WT and *tk1545* KO, generating a ladder of DNA fragments from WT and *tk1545* KO RNAs (Figure 2C, tRNA-Trp) but not when reverse transcriptase was omitted in the reaction (no RT). Out of the 18 candidate circRNAs, 16 were detected circularized in the WT, but not in *tk1545* KO. A circular form of C/D box sR01 and *TK2109* could not be detected in WT or *tk1545* KO (Figure 2C; lanes 3 and 26, respectively), possibly due to a heterogeneous mixture of circular junction sequences in these RNAs. While most of the circular junction sequences represent ligation between the predicted 5′-end and 3′-end, some of the circRNA reads had a few nucleotides missing at the circular junction, which may have affected the PCR amplification step.

### RNA Ligation Activity on C/D Box sRNA

To determine whether Rnl preferentially recognizes C/D box sRNA sequence elements, we assayed for the ligation activity *in vitro* using a 5′-monophosphate terminated C/D box sR42 and sR31 sRNAs as substrates. These two C/D box sRNAs were selected because both are highly enriched and circularized in the WT sample (Table 1; Figure 2C). We previously showed that TkoRnl is capable of circularizing 24-mer single-stranded RNA, but the circularization activity was weak compared to the *M. thermoautotrophicus* enzyme (MthRnl) (Yoshinari et al., 2017; Zhang and Tripathi, 2017); thus, MthRnl was used for the ligation assay. MthRnl is a homolog of TkoRnl (NCBI BLAST E-value of 6 × 10^−60^). The biochemical activity of MthRnl has been extensively characterized and all amino acid residues found to be essential for the MthRnl ligation activity are conserved in TkoRnl (Zhelkovsky and McReynolds, 2014; Torchia et al., 2008; Gu et al., 2016; Yoshinari et al., 2017).

**FIGURE 3:**
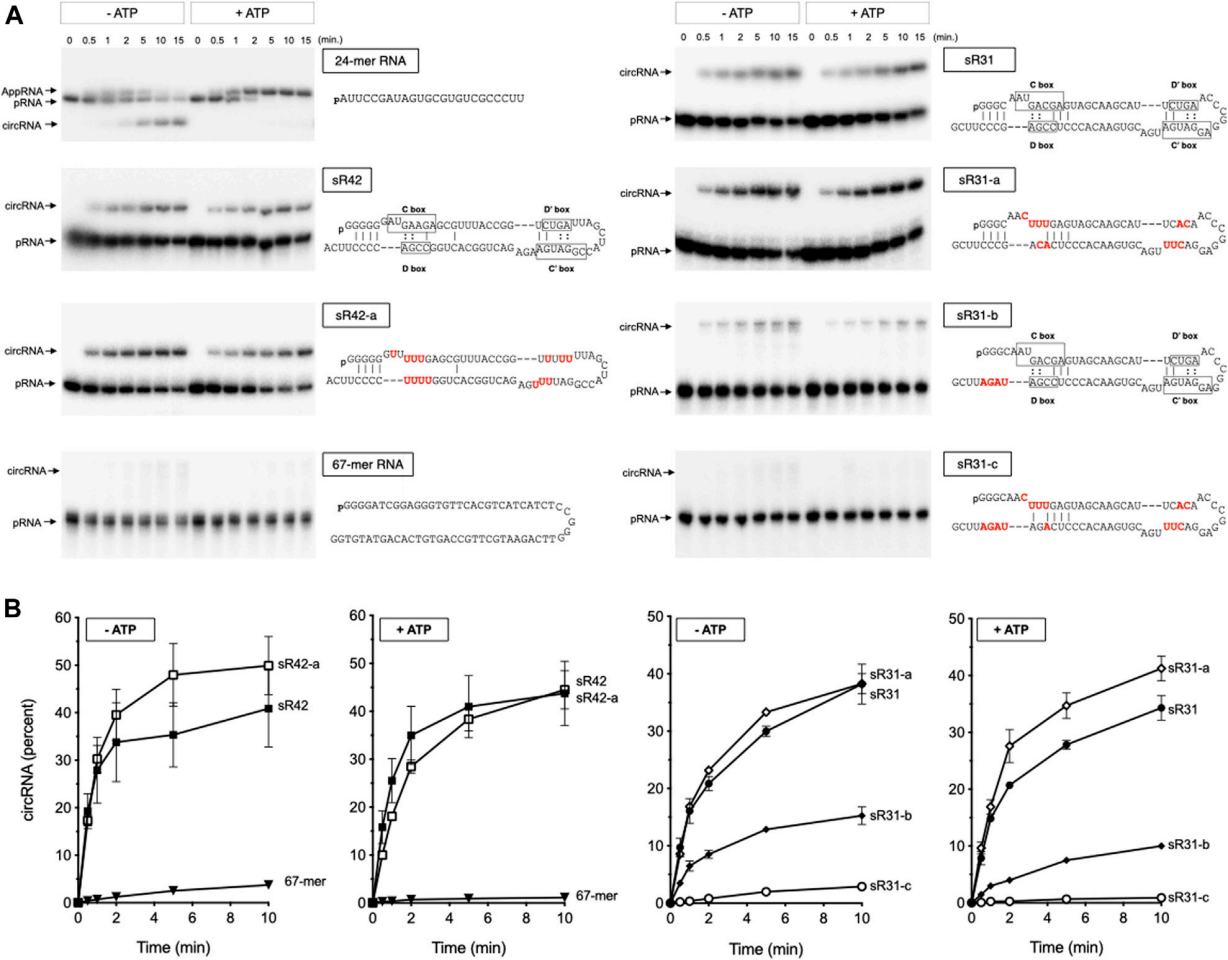
Characterization of RNA circularization activity of MthRnl on C/D box sRNA. **(A)** MthRnl (360 ng) was incubated with 2 pmol of indicated pRNA at 70°C in a reaction mixture (40 μL) that contained 50 mM Tris-HCl (pH 6.5), 1 mM MgCl_2_, in the presence or absence of 1 mM ATP. Aliquots (3 μL) were withdrawn at the times indicated and the products were separated by denaturing polyacrylamide gel. Positions of pRNA, AppRNA, and circRNA are indicated on the left. The sequences of pRNA substrates are illustrated on the right. The conserved C/D box sequence elements in sR42 and sR31 are highlighted, and the variant nucleotides in a mutant form of sR42 and sR31 RNAs are colored in red. **(B)** The yield of circular RNA products by MthRnl in the presence (+ATP) and absence (−ATP) of ATP is plotted as a function of time. The data shown represent the average of three separate experiments with SE bars.

Incubation of MthRnl with either 5′-monophosphate terminated sR42 or sR31 RNAs generates a circRNA molecule that migrates slower than the linear pRNA on a denaturing PAGE (Figure 3A). The circularity of the slower migrating RNA product was verified by its resistance to alkaline phosphatase and RNase R treatment (data not shown). Under identical conditions, MthRnl could not ligate a non-structured 67-mer RNA, suggesting that a structure on the C/D box sRNAs is necessary for RNA circularization. Ligation in the absence of added ATP reflects the presence of pre-adenylated ligase intermediate in the enzyme preparation (Torchia et al., 2008; Gu et al., 2016). We also note that inclusion of ATP in the reaction did not affect circularization of sR42 and sR31 RNAs. This contrasts with ligation of a 24-mer pRNA substrate, which accumulates AppRNA intermediate and suppress the circularization in the presence of ATP, as shown previously for MthRnl (Torchia et al., 2008; Gu et al., 2016), and T4 Rnl2 (Ho and Shuman, 2002; Yin et al., 2003; Nandakumar et al., 2004).

The circularization activity was not significantly affected when the conserved C/D box sequence was substituted with different bases (Figure 3A; sR42-a and sR31-a). However, a mutant form of sR31 RNA that can alleviate hybridization between the terminus was a poor substrate for ligation (Figures 3A,B; sR31-b). Furthermore, MthRnl was inert for circularizing the RNA when both the C/D box and the 3′-terminal sequences were altered (Figures 3A,B; sR31-c). As a control reaction, we showed that bacteriophage T4 Rnl2 could efficiently circularize both the linear 67-mer and sR31-c RNAs (Supplementary Figure S4). We conclude that conserved C/D box sequence elements are not strictly required for circularization by MthRnl. The sequence surrounding the termini could be important for guiding the two termini in close proximity to be recognized by archaea Rnl to allow for intramolecular ligation.

### Other circRNAs in *T. kodakarensis*

It has been widely reported that tRNA introns and rRNA processing intermediates are circularized in various archaea species (Danan et al., 2012; Su et al., 2013; Becker et al., 2017; Jüttner et al., 2019; Qi et al., 2020; Breuer et al., 2021). In both WT and *tk1545* KO, high-levels of circRNA reads were detected from the 16S-23S rRNA operon and tRNA^Trp^ intron (Supplementary Data S2). Inspection of circular junction sequences in total RNA-Seq data reveal that tRNA^Trp^ intron (1,415 reads), 16S circRNA-1 (6,835 reads) and 23S circRNA-1 (282,955 reads) are likely cleaved at the Bulge-Helix-Bulge (BHB) motifs by tRNA splicing endonuclease and joined by tRNA ligase RtcB to form a circular rRNA processing intermediate, as reported (Trotta et al., 1997; Englert et al., 2011; Popow et al., 2011). The circularized tRNA^Trp^ intron was also detected in our RT-PCR analysis in abundance, both in WT and *tk1545* KO (Figure 2C), implying that deletion of TkoRnl does not have an impact on the RtcB ligation pathway.

### Effect on rRNA Processing by Rnl Knock-Out

**FIGURE 4:**
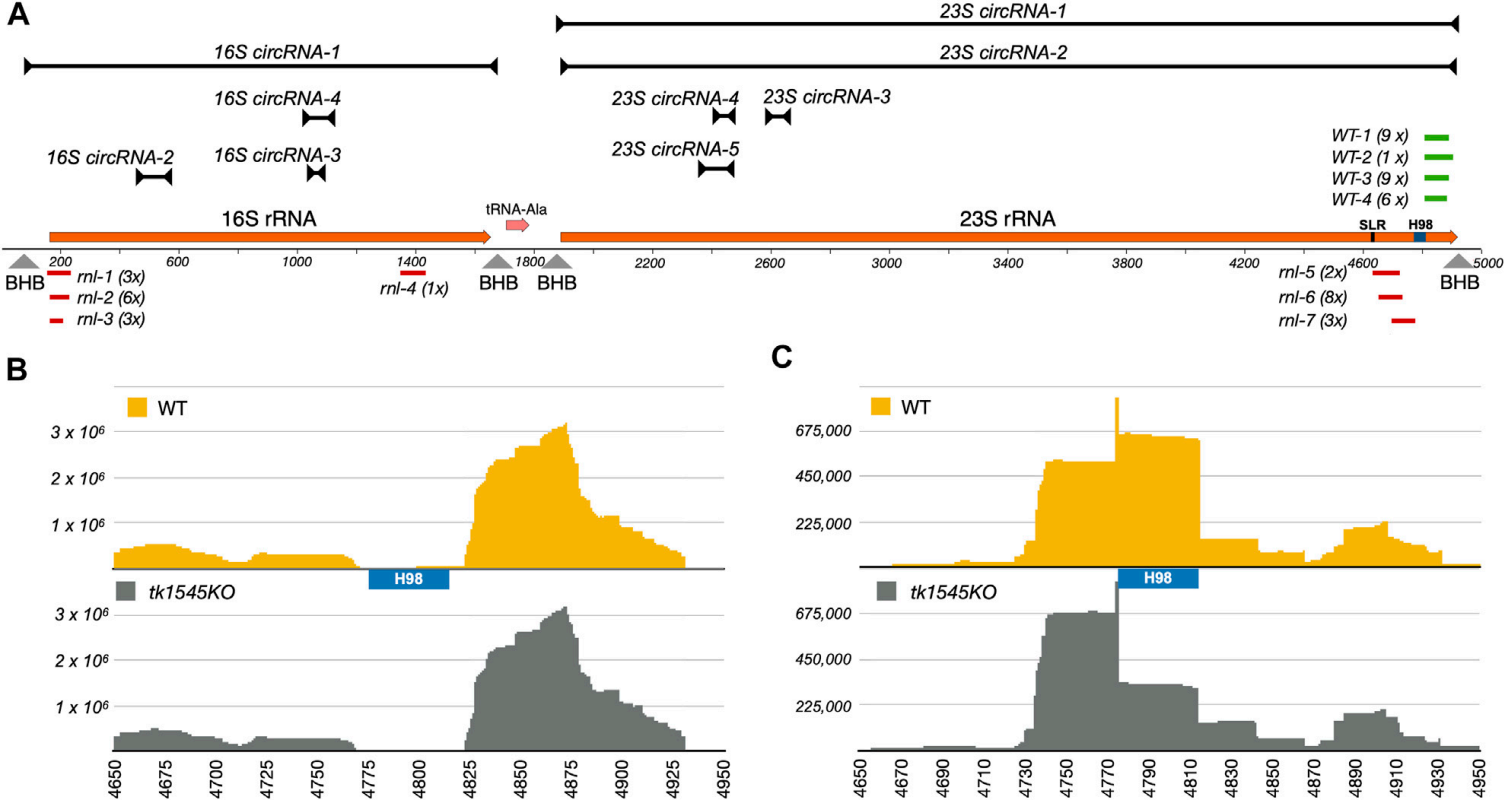
Effect of *TK1545* knockout on rRNA circularization and processing. **(A)** Circular RNAs derived from *T. kodakarensi*s 16S-tRNA^Ala^-23S rRNA operon. A diagram on the bottom represents a 5 kb segment of *T. kodakarensis* 16S-tRNA^Ala^-23S rRNA operon, corresponding to 2022701–2027700 of NC_006624.1 reference sequence. The 16S rRNA, tRNA^Ala^, and 23S rRNA genes are colored in orange. Positions of SRL and H98 are annotated. Arrowheads indicate predicted positions of bulge-helix-bulge motifs (BHB) on the primary transcript. The black lines above the diagram represent the expected size and position of highly represented circRNAs (Supplementary Data S2). Predicted length and position were determined from total RNA-Seq reads containing the junction sequence. Small RNAs fragments derived from WT and *T. kodakarensis* were cloned into TOPO vector via adaptor-mediate RNA ligation. The cloned fragments derived from WT (*WT*: green) or *tk1545* KO (*rnl*: red) were aligned to the reference genome. The number on the right represents number of identical RNA fragments recovered in the RNA cloning experiment (i.e., *2 x indicates twice*). See Supplementary Figure S5 for circular junction and alignment of small RNA fragments with the 16S-tRNA^Ala^-23S rRNA operon. **(B)** Read-coverage in 23S rRNA at the 3′-end from total RNA-Seq dataset from WT (top) and *tk1545* KO (bottom). **(C)** Read-coverage in 23S rRNA at the 3′-end from small RNA-Seq dataset from WT (top) and *tk1545* KO (bottom). The read counts (*y*-axis) for the *tk1545* KO were normalized to the WT read counts. The position of H98 is marked below the WT read-coverage.

In addition to the circular rRNA processing intermediate, we also detected a high level of circular junction reads (42,016 reads) near the predicted 5′-and 3′-ends of 23S rRNA (Figure 4A; 23S circRNA-2). Recent studies in *Pyrococcus furiosus* suggest that 3′-end of 23S rRNA could be fused to the 5′-end by an RNA rearrangement as a consequence of excision of 40-nts helix 98 (H98) located ∼100 nucleotides upstream of the mature 3′-end (Birkedal et al., 2020). Similar to *P. furiosus* rRNA, the H98 could be excised in *T. kodakarensis*, evinced by low coverage of RNA-Seq reads within the equivalent segment (Figure 4B).

We noted earlier that the relative distribution of small RNA species was altered in *tk1545* KO (Figure 1B). While this change is partially attributable to the reduced level of circular C/D box sRNAs in *tk1545* KO, it cannot solely account for the observed differences because a fraction of C/D box sRNAs are likely circularized. We therefore cloned and sequenced the small RNA fragments accumulated in WT and *tk1545* KO. RNA populations in the range of 70–120 nts were isolated from WT and *tk1545* KO cells by gel electrophoresis, annealed to an adapter oligonucleotide containing six degenerate nucleotides at the 3′ end, ligated, and then converted into cDNAs by reverse transcription. The cDNA fragment was cloned into a plasmid using TA cloning and transformed into bacteria. Plasmids isolated from the individual colonies were sequenced using the Sanger method. Note that the adaptor sequence used for RNA cloning has a degenerate sequence at the ends, which allowed us to verify that each obtained clone was derived from an independent RNA and that no single clone was over-represented during PCR amplification.

Alignment of sequences retrieved from small RNA cloning shows that all the fragments obtained were derived from 16S and 23S rRNAs. There were no overlaps between the fragments recovered from WT and *tk1545* KO, indicating that this difference is a consequence of Rnl deletion. Fragments isolated from WT were all derived from 23S rRNA, between the excision site of H98 to the 3′-end of the predicted 23S rRNA (*WT-1* through *WT-4* colored as green in Figure 4A; Supplementary Figure S5). In the *tk1545* KO, nearly half of the cDNA fragments matched the 23S rRNA. Notably, all the fragments mapped upstream of the H98 near the sarcin-ricin loop (SRL) (*rnl-5* through *rnl-7*; colored in red and Supplementary Figure S5). The remaining half of the fragments were derived from 16S rRNA that mapped at the 5′-end of 16S rRNA (*rnl-1* through *rnl-3*; colored in red). Consistent with this finding, analysis of small RNA-Seq data reveals that the reads-coverage upstream of H98 are more abundant in the TkoRnl deletion strain than the WT. (Figure 4C). Taken together, our results suggest that Rnl may participate in rRNA processing, either directly by joining the breaks near the SRL or indirectly through the formation of circular C/D box sRNA.

## Discussion

Here we generated an allelic knock-out of ATP-dependent RNA ligase in *T. kodakarensis* to determine the biological targets of archaeal Rnl. Whereas TkoRnl was not essential for the growth of *T. kodakarensis* under standard laboratory conditions, we showed that its absence abolishes circularization of C/D box sRNAs. The conserved C/D box sequence element, however, was not sufficient for circularization because not all C/D box sRNAs were circularized in *T. kodakarensis.* Furthermore, we demonstrated that recombinant Rnl was capable of forming circular C/D box sRNA with a mutation in the conserved sequence element. The archaeal Rnl could not circularize unstructured RNA of a similar length or C/D box sRNAs that have disrupted terminal stem structures. We conclude that archaeal Rnl may preferentially recognize the terminal stem, and the proximity of the two ends could be critical for intramolecular ligation.

We also detected numerous circular RNA-Seq reads derived from 16S and 23S rRNAs in *T. kodakarensis*. As expected, circularization of tRNA intron or rRNA processing intermediates was not affected in the absence of Rnl, which implies that Rnl does not affect the RtcB ligation pathway. Our finding that C/D box sRNAs are prime substrates for archaeal Rnl is consistent with the previous findings that circular C/D box sRNA-Rnl complexes were detected in *P. abyssi* (Becker et al., 2017).

The functional significance of circular C/D Box sRNA is unclear. C/D box sRNAs have been reported to function as a guide RNA for methylating tRNAs and rRNAs. Circular C/D Box sRNA may alter the specificity to guide RNA to regulate rRNA methylation. However, we did not detect significant differences in the expression, or the overall read coverage, of rRNA genes from the whole transcriptome RNA-seq analysis, between the WT and TkoRnl deletion strains. Comparative transcriptomics analysis revealed that TkoRnl may alter that abundance of subset of C/D Box sRNAs (Figure 2B). TkoRnl could also be involved by regulating the expression of genes involved in sulfur or iron metabolism (Figure 1; Supplementary Table S2). We note that only one biological replicate was analyzed in this study. While it is clear that TkoRnl is responsible for C/D box sRNA circularization, further analysis is necessary to evaluate the biological function of Rnl in archaea.

Similar to *P. furiosus*, *T. kodakarensis* appears to excise H98 from 23S rRNA (Figure 4B), consistent with the finding that the H98 is not present in the cryo-EM structure of *T. kodakarensis* 70S rRNA (Birkedal et al., 2020; Sas-Chen et al., 2020). We found that *T. kodakarensis* accumulates ∼90 nts fragments consisting of a sequence that matches the H98 3′-cleavage site to the predicted 3′-end of 23S rRNA. We speculate that the excision of H98 releases the 3′-end fragment and may have accumulated in *T. kodakarensis*. Intriguingly, we did not retrieve the same fragments from the TkoRnl deletion strain. Instead, we recovered fragments that mapped upstream of H98 near the SRL. SRL interacts with the translational elongation factors that hydrolyze GTP during translocation (Wool et al., 1992; Szewczak et al., 1993; Schmeing et al., 2009), and the cleavage or modification by ribotoxins could block ribosome translocation (Wool et al., 1992; Szewczak et al., 1993; Schmeing et al., 2009).

While it is tempting to speculate that Rnl directly participates in joining the breakage upon excision of H98, we fail to detect any RNA-Seq reads suggesting such “*cis*-splicing” events near the 3′-end of *T. kodakarensis* 23S rRNA. Furthermore, TkoRnl is not likely involved in rearranging the 3′- and 5′-ends of the 23S rRNA as observed in *P. furious* (Birkedal et al., 2020) because permuted reads containing junction sequence between the 5′- and 3′- ends of 23S rRNA were detected in both WT and TkoRnl deletion strain (Table 1; Supplementary Data S2, S3). Therefore, Rnl ligation activity may not act on rRNA directly. It is plausible that Rnl may act indirectly through the formation of circular C/D box sRNA, which in turn could regulate rRNA processing.

Nonetheless, the phylogenetic analysis suggests a possible link between the archaea Rnl, C/D box sRNA circularization, and H98 processing. A high abundance of circular C/D box sRNA molecules was detected in *T. kodakarensis*, *P. furiosus*, *P. abyssi*, *and Methanopyrus kandleri* (Starostina et al., 2004; Danan et al., 2012; Su et al., 2013; Toffano-Nioche et al., 2013). They all encode a homodimeric type-3 Rnl (Gu et al., 2016) and possess H98 or an equivalent structural element in their large subunit of rRNA. In *T. kodakarensis* and *P. furiosus*, H98 is excised evinced by discontinuous RNA-Seq map coverage ((Birkedal et al., 2020) and this study). While many species from *Methanomicrobiales* and *Archaeoglobales* encode Rnl, the helix equivalent of H98 is replaced with a short linker sequence (Birkedal et al., 2020). In contrast, *Haloferax volcanii*, *Nanoarchaeum equitans*, *Sulfolobus solfataricus*, *Sulfolobus acidocaldarius*, and *Pyrobaculum aerophilum*, do not encode homolog of type-3 Rnl. Circular tRNA intron and rRNA intermediates are present in abundance, but only a modest number of circular C/D box RNAs were reported in *H. volcanii*, *S. solfataricu*, *S. acidocaldarius*, and *N. equitans* (Danan et al., 2012; Randau, 2012; Becker et al., 2019). The large subunits of *S. acidocaldarius* and *P. aerophilum* rRNAs were shown to retain H98 evinced by a continuous read coverage at the 3′-end (Birkedal et al., 2020). Because many RNA-Seq data are depleted for rRNA, it is difficult to evaluate its read coverage. Availability of complete RNA-Seq data from other archaea species could provide further insight into the role of Rnl and its relationship to small RNA circularization and rRNA processing.

## Data Availability

The datasets presented in this study can be found in online repositories. The names of the repository/repositories and accession number(s) can be found below: https://www.ncbi.nlm.nih.gov/, GSE186817.

## References

[B1] Abelson J. Trotta C. R. Li H. (1998). tRNA Splicing. J. Biol. Chem. 273, 12685–12688. 10.1074/jbc.273.21.12685 9582290

[B2] Amitsur M. Levitz R. Kaufmann G. (1987). Bacteriophage T4 Anticodon Nuclease, Polynucleotide Kinase and RNA Ligase Reprocess the Host Lysine tRNA. EMBO J. 6, 2499–2503. 10.1002/j.1460-2075.1987.tb02532.x 2444436 PMC553660

[B3] Atomi H. Fukui T. Kanai T. Morikawa M. Imanaka T. (2004). Description ofThermococcus Kodakaraensissp. nov., a Well Studied Hyperthermophilic Archaeon Previously Reported asPyrococcussp. KOD1. Archaea 1, 263–267. 10.1155/2004/204953 15810436 PMC2685570

[B4] Becker H. F. Héliou A. Djaout K. Lestini R. Regnier M. Myllykallio H. (2017). High-throughput Sequencing Reveals Circular Substrates for an Archaeal RNA Ligase. RNA Biol. 14, 1075–1085. 10.1080/15476286.2017.1302640 28277897 PMC5680704

[B5] Becker H. F. L'Hermitte-Stead C. Myllykallio H. (2019). Diversity of Circular RNAs and RNA Ligases in Archaeal Cells. Biochimie 164, 37–44. 10.1016/j.biochi.2019.06.011 31212038

[B6] Birkedal U. Beckert B. Wilson D. N. Nielsen H. (2020). The 23S Ribosomal RNA from Pyrococcus Furiosus Is Circularly Permuted. Front. Microbiol. 11, 582022. 10.3389/fmicb.2020.582022 33362734 PMC7758197

[B7] Brennicke A. Marchfelder A. Binder S. (1999). RNA Editing. Fems Microbiol. Rev. 23, 297–316. 10.1111/j.1574-6976.1999.tb00401.x 10371035

[B8] Breuer R. Gomes-Filho J.-V. Randau L. (2021). Conservation of Archaeal C/D Box sRNA-Guided RNA Modifications. Front. Microbiol. 12, 654029. 10.3389/fmicb.2021.654029 33776983 PMC7994747

[B9] Brooks M. A. Meslet-Cladiére L. Graille M. Kuhn J. Blondeau K. Myllykallio H. (2008). The Structure of an Archaeal Homodimeric Ligase Which Has RNA Circularization Activity. Protein Sci. 17, 1336–1345. 10.1110/ps.035493.108 18511537 PMC2492811

[B10] Burroughs A. M. Aravind L. (2016). RNA Damage in Biological Conflicts and the Diversity of Responding RNA Repair Systems. Nucleic Acids Res. 44, 8525–8555. 10.1093/nar/gkw722 27536007 PMC5062991

[B11] Danan M. Schwartz S. Edelheit S. Sorek R. (2012). Transcriptome-wide Discovery of Circular RNAs in Archaea. Nucleic Acids Res. 40, 3131–3142. 10.1093/nar/gkr1009 22140119 PMC3326292

[B12] Englert M. Beier H. (2005). Plant tRNA Ligases Are Multifunctional Enzymes that Have Diverged in Sequence and Substrate Specificity from RNA Ligases of Other Phylogenetic Origins. Nucleic Acids Res. 33, 388–399. 10.1093/nar/gki174 15653639 PMC546159

[B13] Englert M. Sheppard K. Aslanian A. Yates J. R. Söll D. (2011). Archaeal 3'-phosphate RNA Splicing Ligase Characterization Identifies the Missing Component in tRNA Maturation. Proc. Natl. Acad. Sci. 108, 1290–1295. 10.1073/pnas.1018307108 21209330 PMC3029724

[B14] Gu H. Yoshinari S. Ghosh R. Ignatochkina A. V. Gollnick P. D. Murakami K. S. (2016). Structural and Mutational Analysis of Archaeal ATP-dependent RNA Ligase Identifies Amino Acids Required for RNA Binding and Catalysis. Nucleic Acids Res. 44, 2337–2347. 10.1093/nar/gkw094 26896806 PMC4797309

[B15] Ho C. K. Shuman S. (2002). Bacteriophage T4 RNA Ligase 2 (gp24.1) Exemplifies a Family of RNA Ligases Found in All Phylogenetic Domains. Proc. Natl. Acad. Sci. 99, 12709–12714. 10.1073/pnas.192184699 12228725 PMC130525

[B16] Jäger D. Förstner K. U. Sharma C. M. Santangelo T. J. Reeve J. N. (2014). Primary Transcriptome Map of the Hyperthermophilic Archaeon Thermococcus Kodakarensis. BMC Genomics 15, 684–699. 10.1186/1471-2164-15-684 25127548 PMC4247193

[B17] Jüttner M. Weiß M. Ostheimer N. Reglin C. Kern M. Knüppel R. (2019). A Versatile Cis-Acting Element Reporter System to Study the Function, Maturation and Stability of Ribosomal RNA Mutants in Archaea. Nucleic Acids Res. 48, 2073–2090. 10.1093/nar/gkz1156 PMC7038931 31828323

[B18] Kristensen L. S. Andersen M. S. Stagsted L. V. W. Ebbesen K. K. Hansen T. B. Kjems J. (2019). The Biogenesis, Biology and Characterization of Circular RNAs. Nat. Rev. Genet. 20, 675–691. 10.1038/s41576-019-0158-7 31395983

[B48] Liao Y. Smyth G. K. Shi W. (2014). Featurecounts: An Efficient General Purpose Program For Assigning Sequence Reads To Genomic Features. Bioinformat. 30, 923–930. 10.1093/bioinformatics/btt656 24227677

[B49] Liu Y. (2022). Genetic And Functional Analyses Of Archaeal ATP-Dependent RNA Ligase. Ph.D. thesis Tsukuba (Japan): University of Tsukuba. 10.3389/fmolb.2022.811548 PMC9014305 35445080

[B19] McManus M. T. Shimamura M. Grams J. Hajduk S. L. (2001). Identification of Candidate Mitochondrial RNA Editing Ligases from Trypanosoma Brucei. Rna-A Publ. Rna Soc. 7, 167–175. 10.1017/s1355838201002072 PMC1370075 11233974

[B20] Nandakumar J. Ho C. K. Lima C. D. Shuman S. (2004). RNA Substrate Specificity and Structure-Guided Mutational Analysis of Bacteriophage T4 RNA Ligase 2. J. Biol. Chem. 279, 31337–31347. 10.1074/jbc.m402394200 15084599

[B21] Nandakumar J. Schwer B. Schaffrath R. Shuman S. (2008). RNA Repair: an Antidote to Cytotoxic Eukaryal RNA Damage. Mol. Cel. 31, 278–286. 10.1016/j.molcel.2008.05.019 PMC3289999 18657509

[B22] Omari K. E. Ren J. Bird L. E. Bona M. K. Klarmann G. LeGrice S. F. J. (2006). Molecular Architecture and Ligand Recognition Determinants for T4 RNA Ligase. J. Biol. Chem. 281, 1573–1579. 10.1074/jbc.m509658200 16263720

[B23] Popow J. Englert M. Weitzer S. Schleiffer A. Mierzwa B. Mechtler K. (2011). HSPC117 Is the Essential Subunit of a Human tRNA Splicing Ligase Complex. Science 331, 760–764. 10.1126/science.1197847 21311021

[B24] Qi L. Li J. Jia J. Yue L. Dong X. (2020). Comprehensive Analysis of the Pre-ribosomal RNA Maturation Pathway in a Methanoarchaeon Exposes the Conserved Circularization and Linearization Mode in Archaea. RNA Biol. 17, 1427–1441. 10.1080/15476286.2020.1771946 32449429 PMC7549664

[B25] Randau L. (2012). RNA Processing in the Minimal Organism Nanoarchaeum Equitans. Genome Biol. 13, R63. 10.1186/gb-2012-13-7-r63 22809431 PMC3491384

[B26] Rusché L. N. Huang C. E. Piller K. J. Hemann M. Wirtz E. Sollner-Webb B. (2001). The Two RNA Ligases of the Trypanosoma Brucei RNA Editing Complex: Cloning the Essential Band IV Gene and Identifying the Band V Gene. Mol. Cel. Biol. 21, 979–989. 10.1128/mcb.21.4.979-989.2001 PMC99553 11158286

[B27] Sas-Chen A. Thomas J. M. Matzov D. Taoka M. Nance K. D. Nir R. (2020). Dynamic RNA Acetylation Revealed by Quantitative Cross-Evolutionary Mapping. Nature 583, 638–643. 10.1038/s41586-020-2418-2 32555463 PMC8130014

[B28] Sato T. Fukui T. Atomi H. Imanaka T. (2005). Improved and Versatile Transformation System Allowing Multiple Genetic Manipulations of the Hyperthermophilic Archaeon Thermococcus Kodakaraensis. Appl. Environ. Microbiol. 71, 3889–3899. 10.1128/aem.71.7.3889-3899.2005 16000802 PMC1169065

[B29] Sato T. Fukui T. Atomi H. Imanaka T. (2003a). Targeted Gene Disruption by Homologous Recombination in the Hyperthermophilic Archaeon Thermococcus Kodakaraensis KOD1. J. Bacteriol. 185, 210–220. 10.1128/jb.185.1.210-220.2003 12486058 PMC141832

[B30] Sato T. Fukui T. Atomi H. Imanaka T. (2003b). Targeted Gene Disruption by Homologous Recombination in the Hyperthermophilic Archaeon Thermococcus Kodakaraensis KOD1. J. Bacteriol. 185, 210–220. 10.1128/jb.185.1.210-220.2003 12486058 PMC141832

[B31] Schmeing T. M. Voorhees R. M. Kelley A. C. Gao Y.-G. Murphy F. V. Weir J. R. (2009). The Crystal Structure of the Ribosome Bound to EF-Tu and Aminoacyl-tRNA. Science 326, 688–694. 10.1126/science.1179700 19833920 PMC3763470

[B32] Schnaufer A. Panigrahi A. K. Panicucci B. Igo R. P. Salavati R. Stuart K. (2001). An RNA Ligase Essential for RNA Editing and Survival of the Bloodstream Form of Trypanosoma Brucei. Science 291, 2159–2162. 10.1126/science.1058955 11251122

[B33] Sidrauski C. Cox J. S. Walter P. (1996). tRNA Ligase Is Required for Regulated mRNA Splicing in the Unfolded Protein Response. Cell 87, 405–413. 10.1016/s0092-8674(00)81361-6 8898194

[B34] Starostina N. G. Marshburn S. Johnson L. S. Eddy S. R. Terns R. M. Terns M. P. (2004). Circular Box C/D RNAs in Pyrococcus Furiosus. Proc. Natl. Acad. Sci. 101, 14097–14101. 10.1073/pnas.0403520101 15375211 PMC521125

[B35] Su A. A. H. Tripp V. Randau L. (2013). RNA-seq Analyses Reveal the Order of tRNA Processing Events and the Maturation of C/D Box and CRISPR RNAs in the Hyperthermophile Methanopyrus Kandleri. Nucleic Acids Res. 41, 6250–6258. 10.1093/nar/gkt317 23620296 PMC3695527

[B36] Szewczak A. A. Moore P. B. Chang Y. L. Wool I. G. (1993). The Conformation of the Sarcin/ricin Loop from 28S Ribosomal RNA. Proc. Natl. Acad. Sci. 90, 9581–9585. 10.1073/pnas.90.20.9581 8415744 PMC47613

[B37] Tang T. H. Rozhdestvensky T. S. d’Orval B. C. Bortolin M.-L. Huber H. Charpentier B. (2002). RNomics in Archaea Reveals a Further Link between Splicing of Archaeal Introns and rRNA Processing. Nucleic Acids Res. 30, 921–930. 10.1093/nar/30.4.921 11842103 PMC100335

[B38] Toffano-Nioche C. Ott A. Crozat E. Nguyen A. N. Zytnicki M. Leclerc F. (2013). RNA at 92°C. RNA Biol. 10, 1211–1220. 10.4161/rna.25567 23884177 PMC3849170

[B39] Torchia C. Takagi Y. Ho C. K. (2008). Archaeal RNA Ligase Is a Homodimeric Protein that Catalyzes Intramolecular Ligation of Single-Stranded RNA and DNA. Nucleic Acids Res. 36, 6218–6227. 10.1093/nar/gkn602 18829718 PMC2577357

[B40] Trotta C. R. Miao F. Arn E. A. Stevens S. W. Ho C. K. Rauhut R. (1997). The Yeast tRNA Splicing Endonuclease: A Tetrameric Enzyme with Two Active Site Subunits Homologous to the Archaeal tRNA Endonucleases. Cell 89, 849–858. 10.1016/s0092-8674(00)80270-6 9200603

[B41] Uhlenbeck O. C. Gumport R. I. (1982). 2 T4 RNA Ligase. The Enzymes 15, 31–58. 10.1016/s1874-6047(08)60274-7

[B42] Wang L. K. Nandakumar J. Schwer B. Shuman S. (2007). The C-Terminal Domain of T4 RNA Ligase 1 Confers Specificity for tRNA Repair. Rna 13, 1235–1244. 10.1261/rna.591807 17585047 PMC1924901

[B43] Wool I. G. Glück A. Endo Y. (1992). Ribotoxin Recognition of Ribosomal RNA and a Proposal for the Mechanism of Translocation. Trends Biochem. Sci. 17, 266–269. 10.1016/0968-0004(92)90407-z 1502728

[B44] Yin S. Ho C. K. Shuman S. (2003). Structure-function Analysis of T4 RNA Ligase 2. J. Biol. Chem. 278, 17601–17608. 10.1074/jbc.m300817200 12611899

[B45] Yoshinari S. Liu Y. Gollnick P. Ho C. K. (2017). Cleavage of 3′-terminal Adenosine by Archaeal ATP-dependent RNA Ligase. Sci. Rep. 7, 11662. 10.1038/s41598-017-11693-0 28912583 PMC5599603

[B46] Zhang L. Tripathi A. (2017). Archaeal RNA Ligase from Thermoccocus Kodakarensis for Template Dependent Ligation. RNA Biol. 14, 36–44. 10.1080/15476286.2016.1239688 27715457 PMC5270536

[B47] Zhelkovsky A. M. McReynolds L. A. (2014). Polynucleotide 3′-terminal Phosphate Modifications by RNA and DNA Ligasess. J. Biol. Chem. 289, 33608–33616. 10.1074/jbc.m114.612929 25324547 PMC4246112

