# Supplementary material for: Genetic and Functional Analyses of Archaeal ATP-Dependent RNA Ligase in C/D Box sRNA Circularization and Ribosomal RNA Processing
Source: Front Mol Biosci. 2022 Mar 25;9:811548. doi: 10.3389/fmolb.2022.811548 (PMC9014305; doi:10.3389/fmolb.2022.811548)
Supplement: Supplementary file 1 [file Presentation1.pdf]

Supplementary Materials

Supplementary Data S1. Reads Per Kilo-base per Million mapped reads (RPKM) of individual genes in WT and *tk1545KO T. kodakarensis* from the total and small RNA datasets. Provided as separate excel file.

Supplementary Data S2. Circular RNA analysis from *T. kodakarensis* WT and *tk1545* KO from the total RNA-Seq datasets. Provided as separate excel file.

Supplementary Data S3. Circular RNA analysis from *T. kodakarensis* WT and *tk1545* KO from the small RNA-Seq datasets. Provided as separate excel file.

Supplementary Figure S1. Growth rate of *tk1545* KO strain.

Supplementary Figure S2. Small RNA-Seq analysis from *T. kodakarensis* WT and *tk1545* KO.

Supplementary Figure S3. Secondary structure analysis of terminal stem of circularized RNAs.

Supplementary Figure S4. Ligation activity of bacteriophage T4 Rnl2 on C/D box sRNAs.

Supplementary Figure S5: Analysis of small rRNAs fragments obtained from WT and *tk1545* KO cells.

Supplementary Table S1. List of primers used for RT-PCR.

Supplementary Table S2. List of differentially expressed genes in *tk1545* KO that were identified to show significant change in expression with more than 2-fold change from the total RNA-Seq data set.

Supplementary Table S3. C/D box-like sRNAs encoded by *T. kodakarensis*.

Supplementary Table S4. Analysis of *T. kodakarensis* circRNAs on an ABI/SOLiD sequencing platform.

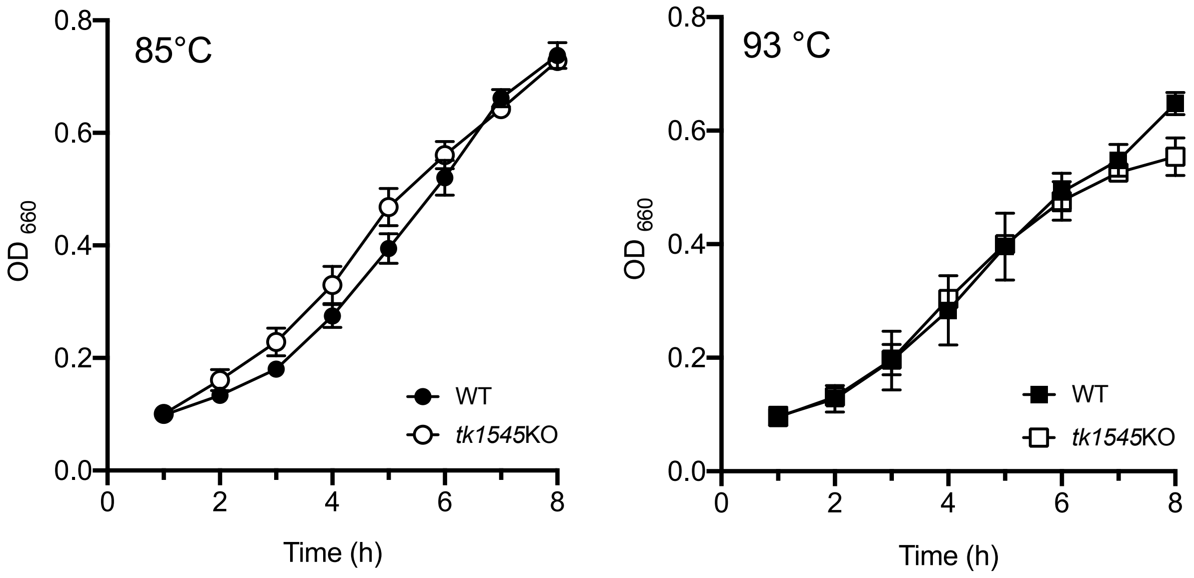

Supplementary Figure S1. Growth rate of *tk1545* KO strain. *T. kodakarensis* strains *KW128* (WT) and *tk1545* KO were pre-cultured in MA-YT-S0 at 85°C for 8 - 12 h, until the absorbance at 660 nm (OD_{660}) reached between 0.3 - 0.5. Cells were then diluted to OD_{660} = 0.0025 in 15 ml of MA-YT-P medium (which lacks elementary sulfur) and cultured at either 85°C (left panel) or 93°C (right panel). Cell density of each strains was measured in parallel at 1 hr intervals after OD_{660} reach 0.1. Error bars indicate the standard deviations of results of triplicate culture experiments.

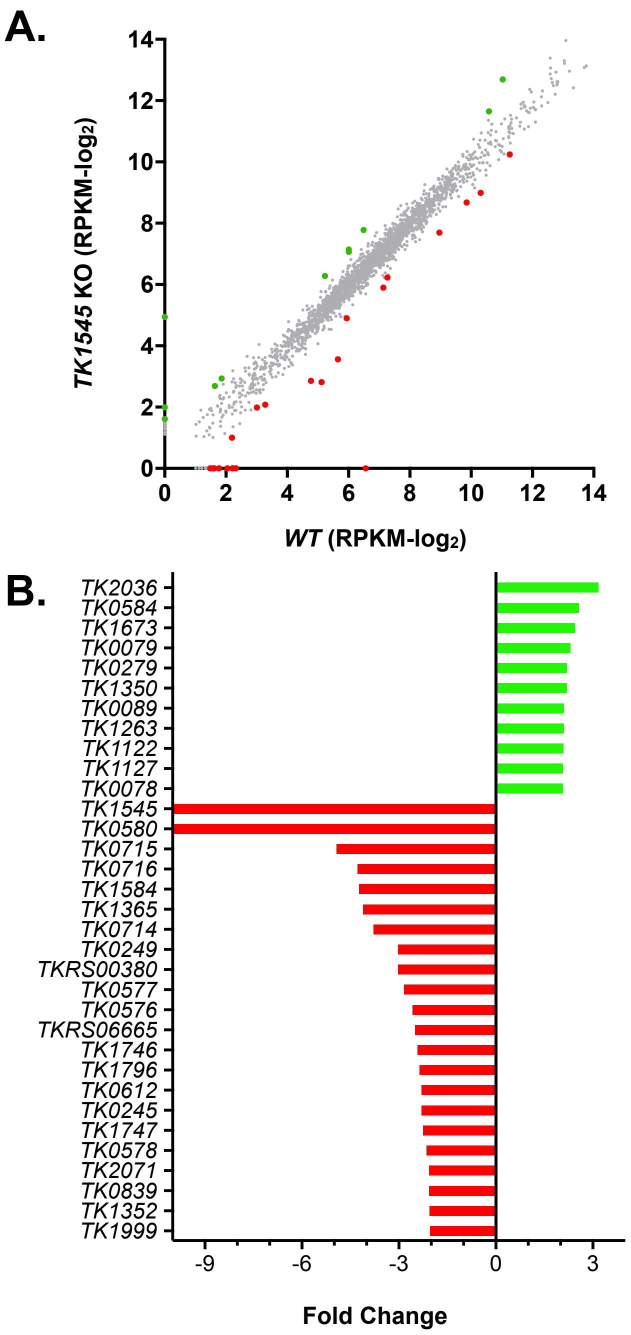

C.

Gene Locus	Gene ID	WT (RPKM)	*tk1545*-KO (RPKM)	Ratio (*tk1545* KO /WT)	Annotated gene name
Up-regulated genes (more than 2- fold):					
TK0078	3235718	3.12	6.43	2.06	Hypothetical membrane protein
TK0079	3234224	1.34	3.07	2.29	Hypothetical membrane protein
TK0089	3234019	3.62	7.65	2.11	Hypothetical protein
TK0279	3235922	64.43	141.81	2.20	Glutamate carrier protein LysW
TK0584	3234263	0.65	1.67	2.57	Hypothetical protein
TK1122	3235586	64.39	134.11	2.08	Prefoldin beta subunit
TK1127	3234655	37.55	77.89	2.07	Hypothetical protein
TK1263	3234901	1529.37	3211.48	2.10	Hypothetical protein
TK1350	3235596	1.82	4.00	2.20	Hypothetical protein
TK1673	3234570	90.09	219.88	2.44	N5-glutamine methyltransferase
TK2036	3235070	2087.15	6605.56	3.16	Permease
Down-regulated genes (more than 2-fold)					
TK0245	3234040	4.60	2.01	0.44	Imidazoleglycerol-phosphate dehydratase
TK0249	3234013	3.08	1.02	0.33	Phosphoribosyl-ATP pyrophosphohydrolase
TK0576	24779727	2.95	1.15	0.39	Hypothetical protein
TK0577	3234072	4.66	1.64	0.35	Hypothetical protein
TK0578	3234073	2.80	1.30	0.46	Hypothetical protein
TK0580	3234223	3.42	0.00	0.00	Hypothetical protein
TK0612	3235396	9.72	4.24	0.44	Hypothetical protein
TK0714	3234907	27.39	7.27	0.27	Iron(II) transport protein B
TK0715	3234908	34.70	7.05	0.20	Iron(II) transport protein A
TK0716	3234532	50.38	11.82	0.23	Iron(II) transport protein A
TK0839	3235916	61.54	29.90	0.49	NAD(P)H-flavin oxidoreductase
TK1352	3233810	8.08	3.97	0.49	Hypothetical protein
TK1365	3233866	4.65	1.13	0.24	Hypothetical protein
*TK1545**	3234155	94.11	0.75	0.01	ATP-dependent RNA ligase (Rnl3)
TK1584	3233730	5.02	1.19	0.24	Hypothetical membrane protein
TK1746	3235478	499.65	206.84	0.41	Hypothetical protein
TK1747	3235479	923.98	409.96	0.44	Inosine/xanthosine triphosphatase
TK1796	3233803	140.36	59.62	0.42	Glutamine synthetase
TK1999	3235059	2456.23	1214.33	0.49	Ferritin-like protein
TK2071	3234523	154.41	75.00	0.49	Sulfhydrogenase subunit gamma
TKRS00380	24779715	4.13	1.37	0.33	Hypothetical protein
TKRS06665	24779745	1270.79	509.83	0.40	tRNA-Leu

Supplementary Figure S2. Small RNA-Seq analysis. WT and *tk1545* KO RNA were subject to small RNA-Seq analysis. Each dot represents an individual gene and are depicted according to RPKM values (**Supplementary Data S1**). Genes with RPKM of < 2.0 in both WT and *tk1545* KO were omitted. Data for C/D box sRNA are presented in Figure 2. Gene that shows greater than two-fold change in *tk1545* KO are colored as green dots (up-regulated) and red dots (down-regulated). Differentially expressed genes are listed in right panel.

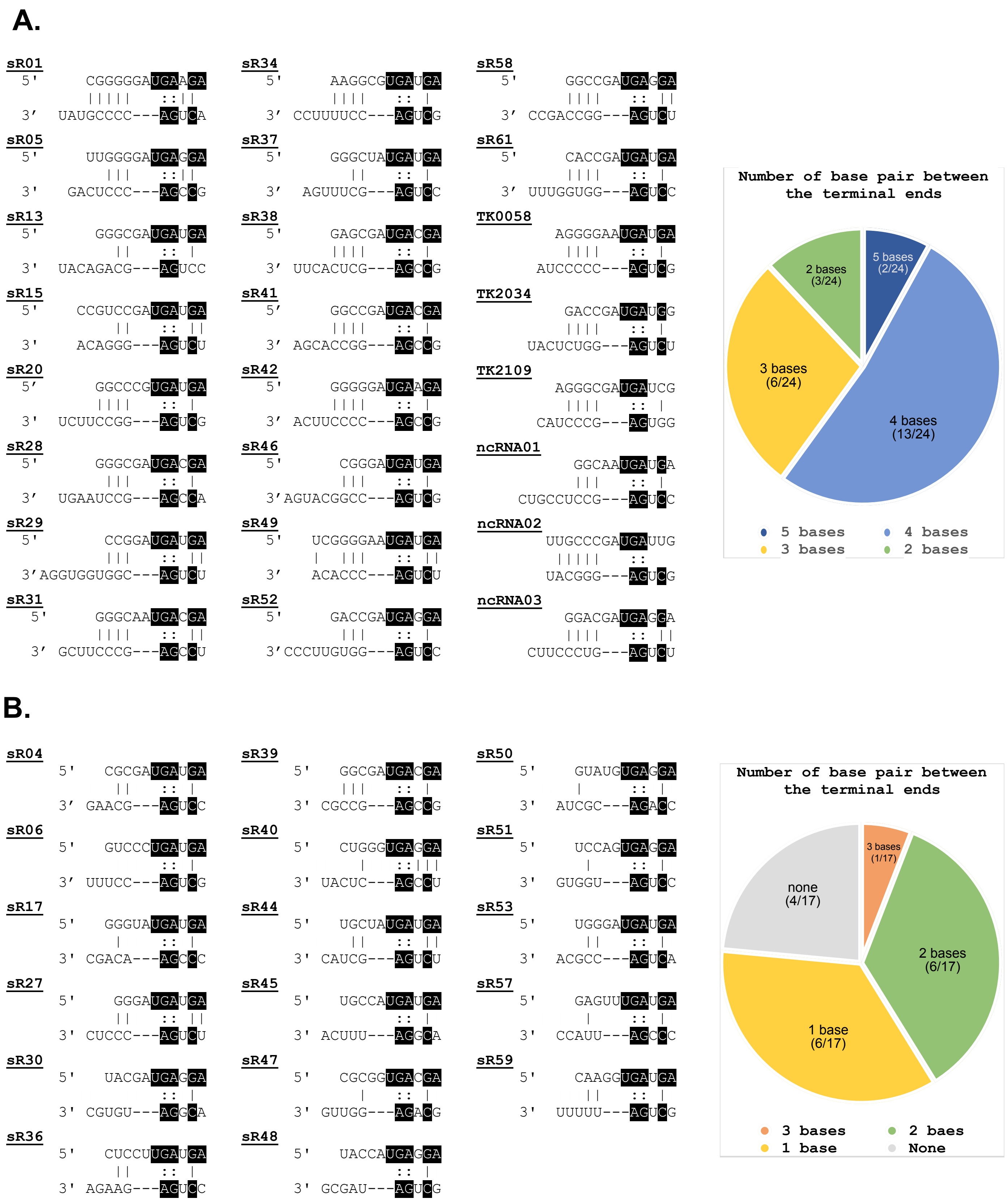

Supplementary Figure S3. Secondary structure analysis of terminal stem of circularized RNAs. Predicted terminal ends of sRNAs from *T. kodakarensis.* List of small RNAs with **(A)** more than twenty circRNA reads or **(B)** without any circRNA reads from WT small RNA-Seq dataset (see **Figure 2 and Supplementary Table S2**). The C/D box sRNAs with less than 1000 aligned reads were omitted for the analysis. C box and D box elements which hybridize to form a K-turn are highlighted. The pie graph on the right represents a distribution of the RNAs with potential number of base pairings in the terminal stem region.

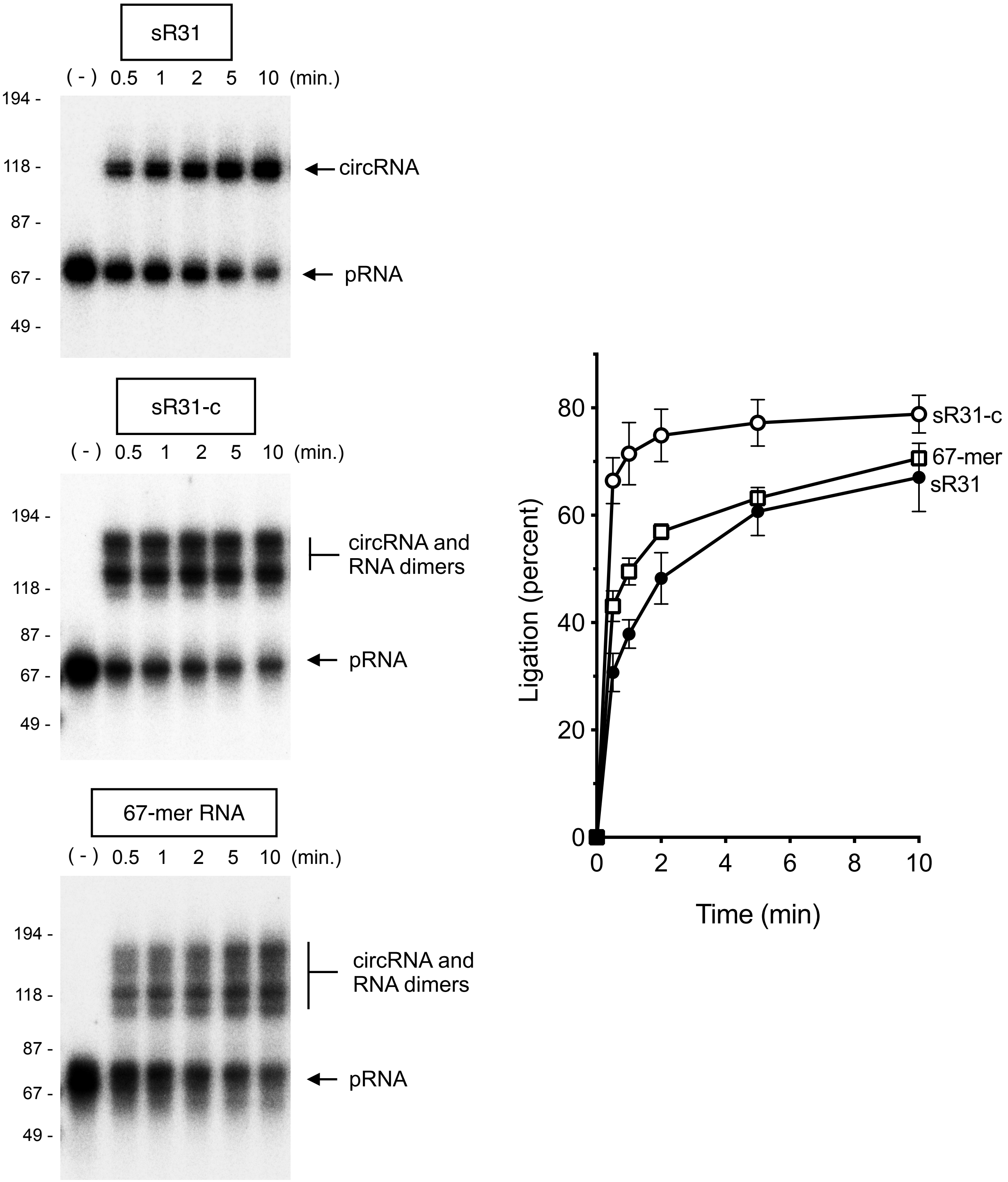

Supplementary Figure S4. Ligation activity of bacteriophage T4 Rnl2 on C/D box sRNAs. Reaction mixture (40 µl) containing 50 mM Tris-HCl (pH 6.5), 5 mM DTT, 1 mM $MgCl_2$, T4 Rnl2 (360 ng), and 2 pmol of either sR-31, sR-31c or 67-mer RNA, were incubated at 22°C. Aliquots (3 µl) were withdrawn at the times indicated. Products were separated by PAGE and visualized by autoradiography. T4Rnl2 is omitted from a control reaction (-). Positions of pRNA, circRNA and possible linear and circular RNA dimer, as observed previously (Ho and Shuman, 2002), are indicated on the right. Percent ligation is plotted as a function of time (*right panel*). The data shown represent the average of three separate experiments with SE bars.

```
4700         10        20        30        40        50        60        70        80        90       100
KOD1-rRNA    AACCCCGCCCTCCAGCTCGGGTCACAAAAAGGTGCGCTCCCGAGGAAACGCCGAAAGGCGGACCCCTCGGGGGTAAGGCAGGCCCGACATCCCGACCAAA
             ~~~~~~~~~~~~~~~~~~~~~~~~~~~~~~~~~~~~~~~~~~~~~~~~~~~~~~~~~~~~~~~~~~~~~~~~~~~~~~~~~~~~~~~~~~~~~~~~

                     110       120       130       140       150       160       170       180       190       200
            5'-end of 16S cirRNA-1  (2022801)
KOD1-rRNA    CCCCGGACGGGAATTATGGTACTTCCCGCTAAGGGAAGTCCCACCGGCCGTACTCCTTGCTCAATTCCGGTTGATCCTGCCGGAGGCCACTGCTATGGGG
             ~~~~~~~~~~~~~~~~~~~~~~~~~~~~~~~~~~~~~~~~~~~~~~~~~~~~~~~~~~~~~~~~~~~~~~~~~~~~~~~~~~~~~~~~~~~~~~~~
                                            (---sR61--)                        (---sR15---)
rnl-1.seq                                                        CCTGCTTAATTCCGGTTGATCCTGCCGGAGGCCCCTGCTATGGGG
rnl-2.seq                                                                aTTCCGGTTGATCCTGCCGGAGGCCACTGCTATGGGG
rnl-3.seq                                                                aTTCCGGTTGATCCTGCCGGAGGCCACTGCTATGGGG

                     210       220       230       240       250       260       270       280       290       300
KOD1-rRNA   GTCCGACTAAGCCATGCGAGTCATGGGGCGCGCTCTGCGCGCACCGGCGGACGGCTCAGTAACACGTCGGTAACCTACCCTCGGGAGGGGGATAACCCCG
             ~~~~~~~~~~~~~~~~~~~~~~~~~~~~~~~~~~~~~~~~~~~~~~~~~~~~~~~~~~~~~~~~~~~~~~~~~~~~~~~~~~~~~~~~~~~~~~~~
rnl-1.seq   GTNCGACTAANCCATGCGAGTCATGGGGCGCG
rnl-2.seq   GTCCGACTAAGCCATGCGAGTCATGGGGCGC
rnl-3.seq   GTCCGACT

                     310       320       330       340       350       360       370       380       390       400
KOD1-rRNA    GGAAACTGGGGCTAATCCCCCATAGGCCTGAGGTACTGGAAGGTCCTCAGGCCGAAAGGGGCATCTGCCCGCCCGAGGATGGGCCGGCGGCCGATTAGGT
             ~~~~~~~~~~~~~~~~~~~~~~~~~~~~~~~~~~~~~~~~~~~~~~~~~~~~~~~~~~~~~~~~~~~~~~~~~~~~~~~~~~~~~~~~~~~~~~~~

                     410       420       430       440       450       460       470       480       490       500
                                                            5'-end of 16S cirRNA-2(2023166)
KOD1-rRNA    AGTTGGTGGGGTAACGGCCCACCAAGCCGAAGATCGGTACGGGCCATGAGAGTGGGAGCCCGGAGATGGACACTGAGACACGGGTCCAGGCCCTACGGGG
             ~~~~~~~~~~~~~~~~~~~~~~~~~~~~~~~~~~~~~~~~~~~~~~~~~~~~~~~~~~~~~~~~~~~~~~~~~~~~~~~~~~~~~~~~~~~~~~~~
                                                                                            (----sR13----)
                     510       520       530       540       550       560       570       580       590       600
                                                                              3'-end of 16S cirRNA-2(2023285)
KOD1-rRNA    CGCAGCAGGCGCGAAACCTCCGCAATGCGGGCAACCGCGACGGGGGGACCCCCAGTGCCGTGGCATAGCCACGGCTTTTCCGGAGTGTAAAAAGCTCCGG
             ~~~~~~~~~~~~~~~~~~~~~~~~~~~~~~~~~~~~~~~~~~~~~~~~~~~~~~~~~~~~~~~~~~~~~~~~~~~~~~~~~~~~~~~~~~~~~~~~
                     (----sR46----)

                     610       620       630       640       650       660       670       680       690       700
KOD1-rRNA    GAATAAGGGCTGGGCAAGGCCGGTGGCAGCCGCCGCGGTAATACCGGCGGCCCGAGTGGTGGCCGCTATTATTGGGCCTAAAGCGTCCGTAGCCGGCCCC
             ~~~~~~~~~~~~~~~~~~~~~~~~~~~~~~~~~~~~~~~~~~~~~~~~~~~~~~~~~~~~~~~~~~~~~~~~~~~~~~~~~~~~~~~~~~~~~~~~
                     710       720       730       740       750       760       770       780       790       800
KOD1-rRNA    GTAAGTCCCTGGCGAAATCCCACGGCTCAACCGTGGGGCTTGCTGGGGATACTGCGGGCCTTGGGACCGGGAGAGGCCGGGGGTACCCCTGGGGTAGGGG
             ~~~~~~~~~~~~~~~~~~~~~~~~~~~~~~~~~~~~~~~~~~~~~~~~~~~~~~~~~~~~~~~~~~~~~~~~~~~~~~~~~~~~~~~~~~~~~~~~
                     810       820       830       840       850       860       870       880       890       900
KOD1-rRNA    TGAAATCCTATAATCCCAGGGGGACCGCCAGTGGCGAAGGCGCCCGGCTGGAACGGGTCCGACGGTGAGGGACGAAGGCCAGGGGAGCGAACCGGATTAG
             ~~~~~~~~~~~~~~~~~~~~~~~~~~~~~~~~~~~~~~~~~~~~~~~~~~~~~~~~~~~~~~~~~~~~~~~~~~~~~~~~~~~~~~~~~~~~~~~~
                     910       920       930       940       950       960       970       980       990      1000
KOD1-rRNA    ATACCCGGGTAGTCCTGGCTGTAAAGGATGCGGGCTAGGTGTCGGGCGAGCTTCGAGCTCGCCCGGTGCCGGAGGGAAGCCGTTAAGCCCGCCGCCTGGG
             ~~~~~~~~~~~~~~~~~~~~~~~~~~~~~~~~~~~~~~~~~~~~~~~~~~~~~~~~~~~~~~~~~~~~~~~~~~~~~~~~~~~~~~~~~~~~~~~~
                     (----sR05---)

                    1010      1020      1030      1040      1050      1060      1070      1080      1090      1100
                 5'-end of 16S cirRNA-4(2023722)
                                                 5'-end of 16S cirRNA-3(2023743)
KOD1-rRNA    GAGTACGGCCGCAAGGCTGAAACTTAAAGGAATTGGCGGGGAGCACTACAAGGGGTGGAGCGTGCGGTTTAATTGGATTCAACGCCGGGAACCTCACCG
             ~~~~~~~~~~~~~~~~~~~~~~~~~~~~~~~~~~~~~~~~~~~~~~~~~~~~~~~~~~~~~~~~~~~~~~~~~~~~~~~~~~~~~~~~~~~~~~~~
                    1110      1120      1130      1140      1150      1160      1170      1180      1190      1200

             3'-end of 16S cirRNA-3(2023804)
                                       3'-end of 16S cirRNA-4(2023831)
KOD1-rRNA    GGGGCGACGGCAGGATGAAGGCCAGGCTGAAGGTCTTGCCGGACACGCCGAGAGGAGGTGCATGGCCGCCGTCAGCTCGTACCGTGAGGCGTCCACTTAA
             ~~~~~~~~~~~~~~~~~~~~~~~~~~~~~~~~~~~~~~~~~~~~~~~~~~~~~~~~~~~~~~~~~~~~~~~~~~~~~~~~~~~~~~~~~~~~~~~~
                    1210      1220      1230      1240      1250      1260      1270      1280      1290      1300
KOD1-rRNA    GTGTGGTAACGAGCGAGACCCGCGCCCCCAGTTGCCAGTCCTCCCCGCTGGGGAGGAGGCACTCTGGGGGGACCGCCGGCGATAAGCCGGAGGAAGGAGC
             ~~~~~~~~~~~~~~~~~~~~~~~~~~~~~~~~~~~~~~~~~~~~~~~~~~~~~~~~~~~~~~~~~~~~~~~~~~~~~~~~~~~~~~~~~~~~~~~~
                                                                                            (----sR58----)

                    1310      1320      1330      1340      1350      1360      1370      1380      1390      1400
KOD1-rRNA    GGGCGACGGTAGGTCAGTATGCCCCGAAACCCCCGGGCTACACGCGCGCTACAATGGGCGGGACAATGGGATCCGACCCCGAAAGGGGAAGGGAATCCCC
             ~~~~~~~~~~~~~~~~~~~~~~~~~~~~~~~~~~~~~~~~~~~~~~~~~~~~~~~~~~~~~~~~~~~~~~~~~~~~~~~~~~~~~~~~~~~~~~~~
rnl-4.seq                                                    gCGACAATGGGCGGGACAATGGGATCCGACCCCGAAAGGGGAAGGGAATCCCC

                    1410      1420      1430      1440      1450      1460      1470      1480      1490      1500
KOD1-rRNA    TAAACCCGCCCTCAGTTCGGATCGCGGGCTGCAACTCGCCCGCGTGAAGCTGGAATCCCTAGTACCCGCGTGTCATCATCGCGCGGCGAATACGTCCCTG
             ~~~~~~~~~~~~~~~~~~~~~~~~~~~~~~~~~~~~~~~~~~~~~~~~~~~~~~~~~~~~~~~~~~~~~~~~~~~~~~~~~~~~~~~~~~~~~~~~
rnl-4.seq    TAAACCCGCCCTCAGTTCGGATCGCGGGCTGCA

                    1510      1520      1530      1540      1550      1560      1570      1580      1590      1600
KOD1-rRNA    CTCCTTGCACACACCGCCCGTCACTCCACCCGAGCGGGGTCCGGGTGAGGCCTGGTCTCCCTTCGGGGAGGCCGGGTCGAGCCTGGGCTCCGTGAGGGGG
             ~~~~~~~~~~~~~~~~~~~~~~~~~~~~~~~~~~~~~~~~~~~~~~~~~~~~~~~~~~~~~~~~~~~~~~~~~~~~~~~~~~~~~~~~~~~~~~~~

                    1610      1620      1630      1640      1650      1660      1670      1680      1690      1700
                                                                              3'-end of 16S cirRNA-1 (2024382)
KOD1-rRNA    GAGAAGTCGTAACAAGGTAGCCGTAGGGGAACCTACGGCTCGATCACCTCCTATCGCCGGAAATCCCGTCCGGGGGGTTTAAGGGATGTCGGGCCTGCCA
             ~~~~~~~~~~~~~~~~~~~~~~~~~~~~~~~~~~~~~~~~~~~~~~~~~~~~~~~~~~~~~~~~~~~~~~~~~~~~~~~~~~~~~~~~~~~~~~~~
             (--sR58-)

                    1710      1720      1730      1740      1750      1760      1770      1780      1790      1800
KOD1-rRNA    TCAGTGGGCCGGTAGCTCAGCCTGGGAGAGCGTCGGCTTTGCAAGCCGAAGGCCCCGGGTTCGAATCCCGGCCGGTCCACCACGAAGAGGTGCACATCCC
             ~~~~~~~~~~~~~~~~~~~~~~~~~~~~~~~~~~~~~~~~~~~~~~~~~~~~~~~~~~~~~~~~~~~~~~~~~~~~~~~~~~~~~~~~~~~~~~~~
```

```
                 1810      1820      1830      1840      1850      1860      1870      1880      1890      1900
                                                                              5'-end of 23S cirRNA-1 (2024584)
                                                                                    5'-end of 23S cirRNA-2 (2024595)
KOD1-rRNA      GAGCACGGCTCGGGGTGGAAGGGCCTGAGACCCCGAACAGGGGTCACGATGAGGGCCGTGCATAGGCGAGCTGGTCCAGAAAACGTCCCGCCCGGTGGCA

                 1910      1920      1930      1940      1950      1960      1970      1980      1990      2000
KOD1-rRNA      ACTAAGCCGCCTGGTGGATGGCTCGGCTCGGGGCGCCGACGAAGGGCGTGGCAAGCTGCGATAAGCCCCGGCGAGGCGCAGGCAGCCGTCGAACCGGGGA
                                                      (---sR01---)                                  (ncRNA01-)

                 2010      2020      2030      2040      2050      2060      2070      2080      2090      2100
KOD1-rRNA      TGCCCGAATGGGACCTCCCGCGGCTTTTGCCGCACTCCCAGTCGGGAGGGGGAACGCGGGGAATTGAAACATCTTAGTACCCGCAGGAAAAGAAAGCAAA
                                                                                                    (---sR01-

                 2110      2120      2130      2140      2150      2160      2170      2180      2190      2200
KOD1-rRNA      AGCGATGCCGTTAGTAGGGGCGACCGAAAGCGGCACAGGGCAAACTGAACCCCGGGCCGAAAGGTTCGGGGATGTGGTGTTGTAGGGCCCGCGGAGGAG
               ---)

                 2210      2220      2230      2240      2250      2260      2270      2280      2290      2300
KOD1-rRNA      CCCTGGGGGCGAAGCCGAAGTCCGCTGGAACGCGGCGCCGGAGAGGGTGAAAGCCCCGTAGGCGTAAGCCCCCGAGCTCCTGCGGTGTCCCTGAGTACCG

                 2310      2320      2330      2340      2350      2360      2370      2380      2390      2400
                          5'-end of 23S cirRNA-5 (2025032)
                                                                 5'-end of 23S cirRNA-4 (2025071)
KOD1-rRNA      TCGGTCGGATATCCGGCGGGAAGCTGGGAGGCATCGGCTCCCAACCCTAAATACGTCCCGAGACCGATAGCGAACTAGTACCGTGAGGGAAAGCTGAAAA
                                                                             (--sR15--)

                 2410      2420      2430      2440      2450      2460      2470      2480      2490      2500
                                            3'-end of 23S cirRNA-4 (2025147)
                                                      3'end of 23S cirRNA-5 (2025151)
KOD1-rRNA      GCACCCCGGGAGGGGGGTGAAAAGAGCCTGAAACCAGGCGGCGATAGGAGGGTGCGGCCCGAAAGGGTTGACCCTCCCCGAAGGAAACACGGGCGACCGT

                 2510      2520      2530      2540      2550      2560      2570      2580      2590      2600
                                                                                    5'-end of 23S cirRNA-3(2025288)
KOD1-rRNA      GGAGTACGAGGGGAGGTGACCGGGGTTGCACCGTCCGTCTTGGATCACGGGGCAGGGAGTTCACCCGAGCGGCGAGGTTAAGGGGGTCAACCCCGAAGCC
                                                                  (----sR58----)

                 2610      2620      2630      2640      2650      2660      2670      2680      2690      2700
                                                                            3'-end of 23S cirRNA-3(2025373)
KOD1-rRNA cl   GCAGGGAAACCGACAGGTCCGCAGCCCGTAAGGGCGAGGGACGGGGTGTGAAAGCGCCCGGAGTCGCTCGGGTGAGACCCGAAGCCGGTCGATCTAGCCC
                                                                                       (---sR20---)
                 2710      2720      2730      2740      2750      2760      2770      2780      2790      2800
KOD1-rRNA cl   GGGGCAGGGTGAAGTCCCTCAACAGAGGGATGGAGGCCCGCTAGGGGTGCTGACGTGCAATTCGCTCCCGTGACCCCGGGCTAGGGGTGAAAGGCCAATC
                                                    (---sR61---)
                                                       (---sR29---)
                 2810      2820      2830      2840      2850      2860      2870      2880      2890      2900
KOD1-rRNA cl   GAGGCCGGCGATAGCTGGTTCCCGCCGAATTATCCCCCAGGATAGCCCGGCCGGAGGTAGGCAGTGGGGTAGAGCACTGATTGGGGGTTTAGGGGGCGAA
                  (---sR29---)          (--sR52--)
                 2910      2920      2930      2940      2950      2960      2970      2980      2990      3000
KOD1-rRNA cl   AGCCTCCGGCTCCCTGTCAAACTCCGAACCCACTGCCGCCGTAGATGGCCGGAGTAGGGGGGCGGTGTAAGCCGTCACCCGAGAGGGGAACAACCCAGAC

                 3010      3020      3030      3040      3050      3060      3070      3080      3090      3100
KOD1-rRNA cl   CGGGGTTAAGGCCCCAAAGTGCCGGCTAAGTGTTACTCCAAAGGGTGTCCCTGGCCTTAGACAGCGGGGAGGTAGGCTTAGAAGCAGCCATCCTTTAAAG

                 3110      3120      3130      3140      3150      3160      3170      3180      3190      3200
KOD1-rRNA cl   AGTGCGTAACAGCTCACCCGTCGAGGTCAGGGGCCCCGAAAATGGACGGGGCTTAAGCCGGCCGCCGAGACCCCGGCGCACGGACCGATTGGTCCGTGAT

                 3210      3220      3230      3240      3250      3260      3270      3280      3290      3300
KOD1-rRNA cl   CGGGTAGGCGGGCGTGCCGATGGGGCGGAAGCCGGGCCGTGAGGTCCGGTGGACCCGTCGGTATTGCGGATCCTGCCGGGAGTAGCAGCATAGCCGGGTG

                 3310      3320      3330      3340      3350      3360      3370      3380      3390      3400
KOD1-rRNA cl   AGAATCCCGGCCGCCGTAGGGGCCAGGGTTCCACGGCAATGTTCGTCAGCCGTGGGTTAGTCGGTCCTAACCCGACCCGTAACTCGGCGTCGGGGAAAGG
                                                                    (--sR49--)
                 3410      3420      3430      3440      3450      3460      3470      3480      3490      3500
KOD1-rRNA cl   GAAACGGGTTTATATTCCCGTACCGCGGTGGTAGGTGCGGCAACGCAAGCCCGAGGGGTGACGCCTCGGGGTAGGCGGACCGGTCCACAAGGCCGGCTAA

                 3510      3520      3530      3540      3550      3560      3570      3580      3590      3600
KOD1-rRNA cl   GCGTATAAGCCCGGGGAGTGCCGTAATGGCGAGAACCGGGTGAAAGCGCGAATGGCCTCCCGTAAGGGGGGTTCCGCCGATCCCTGGGGCCCGTGAAAAG

                 3610      3620      3630      3640      3650      3660      3670      3680      3690      3700
KOD1-rRNA cl   CCCCTCGGGAACGATCCACCGCGACCGTACCGAGAACCGACACTGGTGCCCCTGGGTGAGAAGCCTAAGGCGTGTCGGGGGAAACCCAGCCGAGGGAACT

                 3710      3720      3730      3740      3750      3760      3770      3780      3790      3800
KOD1-rRNA cl   CGGCAAATTGGCCCCGTAACTTCGGGAGAAGGGGTGCCTGCGGGTGTGTAACCCGCAGGTCGCAGTGACTAGGGGGGCCCGACTGTTTAGTAAAAACACA
```

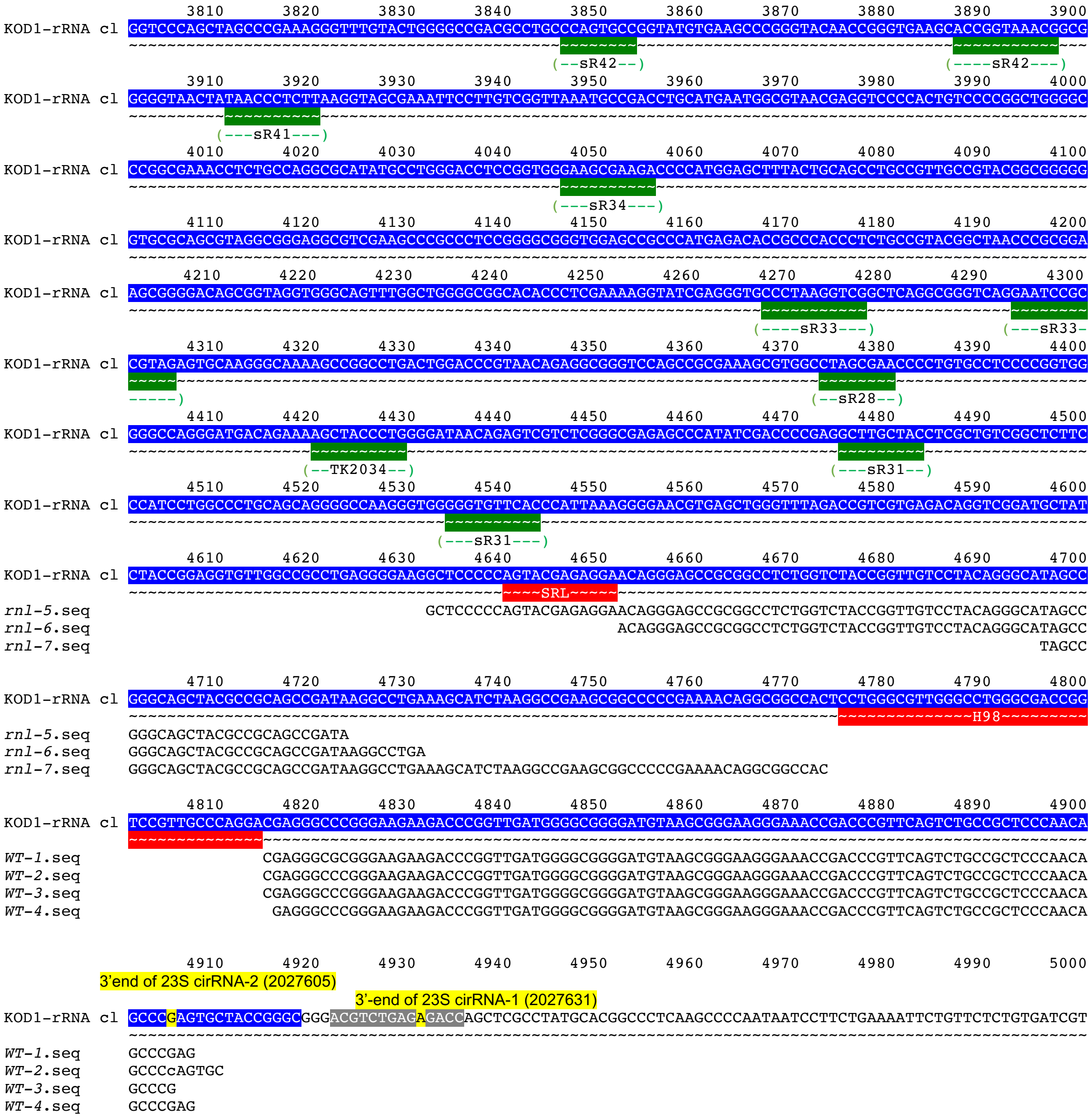

Supplementary Figure S5: Analysis of small rRNAs fragments obtained from WT and *tk1545* KO cells. Small RNA was isolated from WT and *tk1545* KO cells from the gel, converted to cDNA, and cloned into TOPO vector. The sequences of the cloned fragments were aligned to 5 kb segement of *T. kodakarensis* 16S-tRNA^{Ala}-23S rRNA operon (1:5000 corresponding to 2022701:2027700 of NC_006624). The region that corresponds to 16S RNA (red), tRNA-Ala (light green), 23S rRNA (blue) are highlighted. The bulge-helix-bulge motifs are highlighted in gray. Nucleotides highlighted in yellow are the position of circular junction of respective circRNA shown in Figure 4. Predicted helix 98 (H98) and a conserved nucleotides in SRL are highlighted in red. Potential target sequences of the circularized C/D Box guide RNAs in Table 1 are indicated and colored in dark green.

Locus	Gene Specific primer	Circular Junction primer
tRNA-Trp	5'-CTCCAAAGGCTCATCCCCAA	5'-ACCCGGAGAAAGGTATGAGG
sR01	5'-AGGGCGTGCTCATCACAGTC	5'-GACCCCGTATGGGGGATGAA
sR05	5'-GCCCTCCGCTCATCACTTTT	5'-CGACCCTCAGTTGGGGATGA
sR15	5'-AAGACCGATGTCGTCACCGT	5'-AGGGACACCTGTCCGATGAT
sR20	5'-TAGCTGGTGTCCTCACCGCT	5'-GAGGCCTTCTGGCCCGTGAT
sR28	5'-CGAGGGTAGTCATCACCGGT	5'-GAGCCTAAGTGGGCGATGAC
sR31	5'-GTGTTCACGTCATCATCTCCGG	5'-GAGCCCTTCGGGGCAATGAC
sR34	5'-AAGCGAAGAGTCATCACCGC	5'-ACCTTTTCCCAAGGCGTGAT
sR37	5'-CGGCCAAGCTCATCATCCAA	5'-GAGCTTTGAAGGGCTATGAT
sR41	5'-ACCCTCTTTTCCTCATCAGT	5'-GAGGCCACGAGGCCGATGAC
sR42	5'-GTGCCAGTCTCTTCATCCGG	5'-GACCCCTTCAGGGGGATGAA
sR46	5'-CCGCGAAACCTCTTCACCTC	5'-ACCGGCATGAACGGGATGAT
sR49	5'-TCCTCGTCACCGCTCGGAAC	5'-CTGACCCACATCGGGGAATG
sR61	5'-AGTCCCTTCTCATCATCATT	5'-GAGGTGGTTTCACCGATGAT
ncRNA01	5'-CGTCGATCCCGGGCATCCAT	5'-AGCCTCCGTCGGCAATGATG
ncRNA02	5'-AAAGAGAGTGAGCTGAGAGA	5'-CATCGGGCAAATGCCCTCAG
TK0058	5'-GGCAGGGCTCTTCATCGTTT	5'-TGACCCCCTAAGGGGAATGA
TK0705	5'-GGATCCTGGGAAGATGGCTT	5'-CTCCCCTACTTGCCGGAAAC
TK2034	5'-GGAACGGCTTCCACATCAAC	5'-GAGGTCTCATGACCGATGAT
TK2109	5'-GTTCTCCTTTCTGATTTGTG	5'-TCATCGCCCTGTAGGGCTCA

Supplementary Table S1. List of primers used for RT-PCR. The gene specific primer was used for reverse transcriptase reaction. The circular junction primer was added for subsequent PCR.

Gene Locus	Gene ID	WT (RPKM)	*tk1545*-KO (RPKM)	Ratio (*tk1545*-KO/WT)	Annotated gene name
**Up-regulated genes (more than 2- fold):**					
TK0168	3235602	12.75	29.77	2.33	Lrp/AsnC family transcriptional regulator
TK0179	3234083	38.67	82.67	2.14	Hypothetical protein, DUF35 family
TK0180	3234084	44.49	99.58	2.24	Acetyl-CoA acetyltransferase
TK0181	3234194	38.24	85.79	2.24	Hydroxymethylglutaryl-CoA synthase
TK0279	3235922	1.33	3.88	2.92	Lysine biosynthesis protein
TK0546	3233991	5.65	14.57	2.58	Hypothetical protein
TK1463	3234297	5.14	10.40	2.02	Hypothetical protein
TK1673	3234570	3.49	7.56	2.17	N5-glutamine methyltransferase
TK2114	3233929	10.07	20.92	2.08	Hypothetical protein
TK2303	3233720	46.68	109.40	2.34	Chaperonin beta subunit
**Down-regulated genes (more than 2-fold)**					
TK0306	3233840	4.74	2.15	0.45	DEAD/DEAH box RNA helicase
TK0714	3234907	3.75	0.66	0.18	Iron(II) transport protein B
TK0715	3234908	2.61	0.27	0.10	Iron(II) transport protein A
TK0716	3234532	2.79	0.78	0.28	Iron(II) transport protein A
TK1065	3234708	3.13	1.37	0.44	Sulfur-carrier protein
TK1565	3235145	30.42	13.69	0.45	Hypothetical protein
TK1796	3233803	33.49	9.20	0.27	Glutamine synthetase
TK2060	3234926	82.42	26.39	0.32	Phosphate transport system regulator PhoU
TK2061	3234927	72.24	20.60	0.29	Inorganic phosphate transporter, PiT family
TK2070	3234522	61.58	30.05	0.49	Sulfhydrogenase subunit delta
TK2071	3234523	71.84	34.58	0.48	Sulfhydrogenase subunit gamma
TK2072	3234524	68.53	31.49	0.46	Sulfhydrogenase subunit beta
TK2109	3233924	20.74	6.84	0.33	Lipoyl synthase

Supplementary Table S2. List of differentially expressed genes in *tk1545* KO that were identified to show significant change in expression with more than 2-fold change from the total RNA-Seq data set (**Supplementary Data S1**). Gene locus and Gene ID are in accordance to *T. kodakarensis* reference genome (NC_006624.1) and annotated gene names are assigned from KEGG: Kyoto Encyclopedia of Genes and Genomes.

T. kodakarensis C/D box-like sRNA	Start and end (+/- 5 nucleotides)			Predicted length (nts)	*T. kodakarensis* (WT) Number of aligned read	*T. kodakarensis* (WT) Number of circular reads	*T. kodakarensis* (*tk1545* KO) Number of aligned read	*T. kodakarensis* (*tk1545* KO) Number of circular reads	Alias and predicted transcription start site⁺		*P. abyssi* C/D Box sRNA homologs	*P. abyssi* Number of aligned read	*P. abyssi* Number of circular reads
sR01	47786	to	47847	62	2,167	395	1,776	7	TKOcandSno19	47910	Pab-sR09	0	9
sR02	54779	to	54838	60	253	0	140	0					
sR03	58300	to	58357	58	117,134	1	98,318	0	Tko-sR03	58356	Pab-sR29*	0	300
sR04	87729	to	87784	56	146,490	0	82,694	0	Tko-sR04	87730	Pab-sR46*	2,590	236
sR05	116401	to	116466	66	445,142	354	333,035	0	Tko-sR07	116468	Pab-sR18	191	0
sR06	159451	to	159515	65	51,789	0	28,668	0	Tko-sR11	159453	Pab-sR15	0	0
sR07	162929	to	162999	71	314	0	201	0					
sR08	178215	to	178280	66	171	0	124	0					
sR09	206252	to	206314	63	18	0	23	0					
sR10	254603	to	254660	58	13,578	2	7,752	0	Tko-sR12	254604	Pab-sR04*	0	10
sR11	268795	to	268861	67	185	0	131	0					
sR12	274087	to	274147	61	7,484	12	6,249	0	Tko-sR13	274205	Pab-sR10	0	0
sR13	279797	to	279863	67	59,144	47	58,048	0	Tko-sR14	279865			
sR14	287655	to	287727	73	16,443	5	4,240	0	Tko-sR15	287755			
sR15	316130	to	316191	62	1,629,229	698	1,523,609	8	Tko-sR16	316202	Pab-sR14*	0	95
sR16	368491	to	368563	73	777	0	459	0					
sR17	493594	to	493681	88	5,810	0	3,877	0					
sR18	534297	to	534365	69	588	0	317	0					
sR19	545080	to	545151	72	173	0	154	0					
sR20	558818	to	558879	62	1,622	21	1,400	0	Tko-sR20	558914	Pab-sR28	1,024	0
sR21	599335	to	599401	67	79	0	55	0					
sR22	605281	to	605338	58	201	0	172	0					
sR23	617316	to	617387	72	165	0	130	0					
sR24	621932	to	621995	64	588	0	389	0					
sR25	654868	to	654934	67	387	0	215	0					
sR26	669805	to	669874	70	42	0	53	0					
sR27	795506	to	795566	61	107,342	0	53,467	0	Tko-sR24	795566	Pab-sR02*	1,306	66
sR28	832364	to	832425	62	65,322	72	54,801	1	Tko-sR26	832424	Pab-sR39*	0	11
sR29	940146	to	940209	64	475,600	36	283,215	0	Tko-sR29	940134	Pab-sR07*	0	1
sR30	942666	to	942747	82	3,044	0	2,747	0					
sR31	963853	to	963919	67	562,648	4,655	721,884	34	Tko-sR31	963842	Pab-sR03*	0	28
sR32	1001641	to	1001710	70	292	0	264	0					
sR33	1100949	to	1101011	63	170,549	20	97,779	0	Tko-sR34	1101012	Pab-sR13*	3,727	120
sR34	1103565	to	1103626	62	20,161	119	19,347	0	Tko-sR35	1103625	Pab-sR45*	584	87
sR35	1108431	to	1108492	62	550,108	8	209,674	1	Tko-sR36	1108387	Pab-sR31*	483	287
sR36	1133458	to	1133521	64	2,487	0	1,500	0					
sR37	1159583	to	1159644	62	356,961	44	195,173	1	Tko-sR37	1159732	Pab-sR32*	543	35
sR38	1167276	to	1167338	63	11,287	106	4,940	0					
sR39	1167372	to	1167429	58	26,514	0	26,153	0	Tko-sR39	1167431	Pab-sR51	0	3
sR40	1183980	to	1184044	65	2,773	0	2,623	0					
sR41	1226838	to	1226899	62	1,967	31	610	0	Tko-sR41	1226903	Pab-sR34*	0	2,621
sR42	1226948	to	1227017	70	12,085	7,194	17,658	10	Tko-sR42	1226948	Pab-sR12*	1,840	122
sR43	1365876	to	1365945	70	148	0	92	0					
sR44	1368418	to	1368475	58	32,410	0	34,627	0	Tko-sR49	1368474	Pab-sR21*	664	49
sR45	1369006	to	1369070	65	1,385	0	1,076	0					
sR46	1371729	to	1371790	62	176,232	188	79,569	3	Tko-sR50	1371720	Pab-sR23	0	0
sR47	1400539	to	1400606	68	1,603	0	1,156	0					
sR48	1445711	to	1445786	76	1,874	0	2,488	0					
sR49	1446209	to	1446268	60	62,328	358	36,039	1	Tko-sR52	1446266	Pab-sR11*	555	46
sR50	1452262	to	1452324	63	2,020	0	1,736	0					
sR51	1465355	to	1465424	70	1,560	0	1,280	0					
sR52	1476851	to	1476917	67	28,701	467	42,613	6	Tko-sR54	1476850	Pab-sR55*	1,121	44
sR53	1626302	to	1626359	58	208,776	0	150,510	0	Tko-sR57	1626358	Pab-sR60*	18,423	6,365
sR54	1626388	to	1626447	60	157,804	4	43,537	0	Tko-sR58	1626445	Pab-sR26*	10,252	3,087
sR55	1726020	to	1726083	64	1,429,614	6	1,478,232	3	Tko-sR61	1726020	Pab-sR44*	595	41
sR56	1766626	to	1766698	73	615	0	407	0					
sR57	1787986	to	1788070	85	2,302	0	1,285	0	TKOcandSno104	1780963			
sR58	1947796	to	1947856	61	926	95	845	0			Pab-sR25*	2,153	447
sR59	2002453	to	2002515	63	198,717	0	113,994	0	Tko-sR66	2002525	Pab-sR05	95	1
sR60	2062992	to	2063053	62	712	0	419	0					
sR61	2070055	to	2070116	62	100,212	92	31,284	0	Tko-sR67	2070114	Pab-sR52	176	0

Supplementary Table S3. C/D box-like sRNAs encoded by *T. kodakarensis*. A total of sixty-one putative C/D box sRNAs were identified from searching the *T. kodakarensis* reference genome (Genebank NC_006624.1) for C/D box motif. CircRNAs were identified from the WT and *tk1545* KO small RNA-Seq data sets. Some of the circRNA reads had up to 5 nucleotides missing from the end or at the circular junction. The alias and predicted start site of transcription are from (Jäger et al., 2014). CircRNA-Seq analysis on *P.abyssi* RNA-Seq data set (Toffano-Nioche et al., 2013; GSM1401488) was performed and the number of C/D box sRNAs aligned reads and circRNA reads were determined. The nomenclature of *P. abyssi* C/D box sRNA were adopted from (Becker et al., 2017) and C/D box sRNAs which were reported to be circularized are indicated by asterisks.

	Illumina dataset (from Table 1)				SOLiD small RNA-Seq dataset 1				SOLiD small RNA-Seq dataset 2			
	T. kodakarensis (WT)		*T. kodakarensis* (*tk1545* KO)		*T. kodakarensis* (WT)		*T. kodakarensis* (*tk1545* KO)		*T. kodakarensis* (WT)		*T. kodakarensis* (*tk1545* KO)	
Locus / CircRNA name	RPKM aligned read	RPKM circular read	RPKM aligned read	RPKM circular read	RPKM aligned read	RPKM circular read	RPKM aligned read	RPKM circular read	RPKM aligned read	RPKM circular read	RPKM aligned read	RPKM circular read
sR01	212.8	39.4	200.3	0.8	13,051.6	65.6	9,265.3	0.0	13,461.4	85.2	10,608.5	1.5
sR05	40,401.3	35.3	34,717.0	0.0	3,654.2	50.2	4,740.6	0.0	4,124.4	46.8	3,440.2	1.5
sR13	5,287.8	4.7	5,960.9	0.0	1,476.2	24.1	1,757.9	0.0	1,950.1	25.1	2,283.8	0.0
sR15	157,409.4	69.7	169,074.4	0.9	99,416.6	834.0	245,412.3	9.0	135,113.7	841.7	268,802.4	4.5
sR20	156.7	2.1	155.4	0.0	818.6	1,023.2	1,588.2	0.0	2,024.9	1,204.1	2,026.0	3.0
sR28	6,311.1	7.2	6,081.3	0.1	3,909.8	112.9	3,636.8	0.0	4,483.9	145.3	3,766.8	1.5
sR29	46,703.8	3.6	31,943.5	0.0	5,354.8	41.5	7,684.5	7.8	6,407.5	53.4	8,489.8	9.1
sR31	50,304.0	464.7	74,129.1	3.9	15,632.3	419.9	31,288.8	5.2	17,365.0	502.7	31,803.1	9.1
sR34	1,947.9	11.9	101,282.5	0.1	536.0	1.9	485.8	0.0	743.2	30.1	567.8	0.0
sR37	34,488.1	4.4	21,658.3	0.1	309.7	3.9	308.3	0.0	208.8	5.0	388.5	0.0
sR38	1,073.2	10.6	539.5	0.0	3,911.0	312.8	3,734.9	0.0	3,512.3	297.3	3,513.4	0.0
sR41	190.0	3.1	67.7	0.0	2,141.5	205.6	1,514.9	0.0	2,350.5	215.4	1,535.3	1.5
sR42	1,034.2	718.2	1,735.6	1.1	14,684.6	840.8	22,989.6	2.6	16,401.8	945.2	21,267.2	0.0
sR46	16,756.5	18.8	8,689.6	0.3	5,365.3	422.8	4,827.2	2.6	6,090.5	496.0	4,638.3	0.0
sR49	6,222.6	35.7	4,132.5	0.1	3,301.7	91.7	2,829.8	2.6	2,015.7	111.9	1,055.1	0.0
sR52	2,773.0	46.6	4,728.8	0.7	115,289.3	262.6	165,785.0	7.8	158,209.2	405.8	182,679.9	15.1
sR58	90.9	9.5	95.3	0.0	86.4	7.7	90.8	0.0	75.4	11.7	102.5	0.0
sR61	9,682.1	9.2	3,471.6	0.0	2,028.7	790.6	866.4	1.3	1,698.0	1,095.6	1,019.8	3.0
ncRNA01	126,590.9	6.8	67,320.5	0.3	104,085.9	56.0	98,215.4	40.1	137,796.2	66.8	108,032.8	30.3
ncRNA02	803.8	13.9	703.8	0.0	28,622.4	0.0	43,833.6	0.0	35,074.6	8.4	38,178.2	0.0
ncRNA03	638.2	40.2	933.5	0.1	1,056.0	61.8	1,897.5	2.6	1,199.6	38.4	2,470.5	0.0
TK0058	162.4	58.8	80.1	0.1	3,495.3	20,502.8	1,853.4	51.7	4,087.0	18,856.5	1,934.7	45.4
TK2034	70.0	6.6	49.7	0.2	2,965.7	16.4	9,579.0	0.0	3,023.6	20.0	10,668.1	0.0
TK2109	1,398.4	2.8	1,255.8	0.0	2,110.4	330.1	3,048.1	1.3	1,163.1	643.0	2,619.8	0.0
tRNA-Trp	2,792.9	202.0	2,569.6	148.2	10,630.5	368.7	18,133.9	697.9	15,068.7	679.7	18,339.5	713.0

Supplementary Table S4. Analysis of *T. kodakarensis* circRNAs on an ABI/SOLiD sequencing platform. Small RNA libraries were prepared from the WT and *tk1545* KO cells using NEBNext Multiplex Small RNA Library Prep Set for SOLiD (New England Biolabs). The high-throughput RNA-sequencing was performed on ABI/SOLiD platform (35-base read length). The aligned and circular reads (in RPKM) are listed under SOLiD RNA-Seq datasets 1 and 2 (Liu, Y, 2022), along with the data from Illumina small RNA-Seq dataset from **Table 1**.
